# Review: the energetic value of zooplankton and nekton species of the Southern Ocean

**DOI:** 10.1007/s00227-018-3386-z

**Published:** 2018-07-18

**Authors:** Fokje L. Schaafsma, Yves Cherel, Hauke Flores, Jan Andries van Franeker, Mary-Anne Lea, Ben Raymond, Anton P. van de Putte

**Affiliations:** 1 Wageningen Marine Research, Ankerpark 27, 1781 AG Den Helder, The Netherlands; 2 Centre d’Etudes Biologiques de Chizé, UMR 7372 du CNRS et de l’Université de La Rochelle, 79360 Villiers-en-Bois, France; 3 Alfred-Wegener-Institut Helmholtz-Zentrum für Polar-und Meeresforschung, Am Handeshafen 12, 27570 Bremerhaven, Germany; 4 Institute for Marine and Antarctic Studies, University of Tasmania, 20 Castray Esplanade, Battery Point, Hobart, TAS 7004 Australia; 5 Australian Antarctic Division, Department of the Environment and Energy, 203 Channel Highway, Kingston, TAS 7050 Australia; 6 Antarctic and Climate Ecosystems Cooperative Research Centre, University of Tasmania, Private Bag 80, Hobart, TAS 7001 Australia; 7 Royal Belgian Institute of Natural Sciences, Vautierstraat 29, 1000 Brussels, Belgium

## Abstract

Understanding the energy flux through food webs is important for estimating the capacity of marine ecosystems to support stocks of living resources. The energy density of species involved in trophic energy transfer has been measured in a large number of small studies, scattered over a 40-year publication record. Here, we reviewed energy density records of Southern Ocean zooplankton, nekton and several benthic taxa, including previously unpublished data. Comparing measured taxa, energy densities were highest in myctophid fishes (ranging from 17.1 to 39.3 kJ g^−1^ DW), intermediate in crustaceans (7.1 to 25.3 kJ g^−1^ DW), squid (16.2 to 24.0 kJ g^−1^ DW) and other fish families (14.8 to 29.9 kJ g^−1^ DW), and lowest in jelly fish (10.8 to 18.0 kJ g^−1^ DW), polychaetes (9.2 to 14.2 kJ g^−1^ DW) and chaetognaths (5.0–11.7 kJ g^−1^ DW). Data reveals differences in energy density within and between species related to size, age and other life cycle parameters. Important taxa in Antarctic food webs, such as copepods, squid and small euphausiids, remain under-sampled. The variability in energy density of *Electrona antarctica* was likely regional rather than seasonal, although for many species with limited data it remains difficult to disentangle regional and seasonal variability. Models are provided to estimate energy density more quickly using a species’ physical parameters. It will become increasingly important to close knowledge gaps to improve the ability of bioenergetic and food web models to predict changes in the capacity of Antarctic ecosystems to support marine life.

**Electronic supplementary material:**

The online version of this article (10.1007/s00227-018-3386-z) contains supplementary material, which is available to authorized users.

## Introduction

The Southern Ocean is home to some of the largest populations of top predator species worldwide such as penguins, flying birds, seals and whales. It comprises the sub-Antarctic and Antarctic regions and is here defined as the water masses south of the Subtropical Front (STF), which separates the surface waters of the Southern Ocean from the warmer and more saline surface waters of subtropical circulations (Orsi et al. 1995; Belkin and Gordon 1996). To predict consequences of challenges to top predators, such as from climate change and increased fisheries, and to develop adequate conservation measures, a quantitative understanding of the energy flux in the ecosystem is important. The energy content of species is a key factor in models of energy flux in food webs and in the studies of trophic relationships between species (Van de Putte et al. 2006).

The life cycle and physiology of a species can strongly influence its energetic value. Organisms often have seasonal cycles in lipid content and consequently energy density (Hislop et al. 1991; Tierney et al. 2002). This is generally associated with the annual reproductive and feeding cycles (Hislop et al. 1991). Many species, for instance, acquire energy for reproduction, and therefore, have a high energy value just before spawning, and a lower one afterwards (Norrbin and Båmstedt 1984; Van de Putte et al. 2006; Fenaughty et al. 2008). Particularly in crustaceans, energy densities can vary between sexes (Färber-Lorda et al. 2009a). Lipid storage is used as buoyancy control in many marine animals, causing differences in energy content between animals with a different vertical distribution (Lawrence 1976). Furthermore, lipid content changes with size and age, greatly influencing energy content (Tierney et al. 2002; Färber-Lorda et al. 2009a; Färber-Lorda and Mayzaud 2010). Energy allocation for different purposes, such as growth or reproduction, most likely occurs simultaneously, but one purpose may dominate over others depending on locality and season (Båmstedt 1986).

Within a single species, the energetic value can vary between regions or seasons, due to differences in the type or amount of food (Williams and Robins 1979; Tierney et al. 2002; Van de Putte et al. 2006). Temperature and changes in food can, furthermore, influence the energy storage function of prey species (Ruck et al. 2014). Specifically at higher latitudes, the Southern Ocean experiences strong seasonality, with drastic changes in light availability between seasons and massive changes in sea-ice cover in many parts. In winter, the phytoplankton growth in the water column of both ice-covered and open water is greatly reduced (Arrigo et al. 1998, 2008). In ice-covered waters, algae and other fauna within and at the underside of the sea ice may provide the only source of primary production (Eicken 1992; Quetin and Ross 2003; Arrigo et al. 2008; Flores et al. 2011, 2012; Meiners et al. 2012; Schaafsma et al. 2017). A patchy and seasonally changing food distribution can cause frequent periods of starvation. Therefore, organisms living in harsher environment tend to have higher energy content, as they have adapted to the lower degree of predictability of food availability, and energy content and lipid stores of organisms tend to increase towards higher latitudes (Norrbin and Båmstedt 1984; Falk-Petersen et al. 2000).

The winter food scarcity has resulted in different overwintering strategies used by zooplankton and nekton living in the Southern Ocean such as relying on lipids reserves, reducing metabolic activity, dormancy, feeding on sea-ice resources, opportunistic feeding, combustion of tissue, or a combination of these (Torres et al. 1994; Schnack-Schiel et al. 1998; Meyer et al. 2009; Kohlbach et al. 2017; Schaafsma et al. 2017). Species need to make optimal use of periods of high production, for instance to “fatten up” for winter and/or to gain enough energy for reproduction. Timing of reproduction can be important to ensure winter survival of young stages. Many species, therefore, have a specific strategy to make optimal use of spring phytoplankton blooms, which in ice-covered waters is initiated by sea-ice melt, or the peak summer phytoplankton production during their life cycle (Quetin and Ross 1991; Lizotte 2001).

The overwintering strategy utilized by zooplankton and nekton influences its seasonal physiology and consequently, energetic density. Species relying on reserves in winter often have a low energetic value by the end of this season (Torres et al. 1994). Organisms that have accumulated lipids for a time of low phytoplankton availability have relatively high lipid content and high energetic values. Therefore, higher energetic values are often found in herbivores in certain seasons (Donnelly et al. 1994). Species can also have a ‘business as usual’ overwintering strategy, encompassing opportunistic feeding combined with some combustion of tissue (Torres et al. 1994). This strategy is, for instance, adopted by deeper living zooplanktivorous species which do not necessarily experience a food decline during the winter months, as they have access to, e.g. calanoid copepods that sink out of the euphotic zone to overwinter in diapause (Bathmann et al. 1993; Torres et al. 1994; Kruse et al. 2010). Many larger crustaceans adopt a mixed strategy comprising a combination of opportunistic feeding, combustion of body mass, a lowered metabolic rate, and occasionally, negative growth (Ikeda and Dixon 1982; Quetin and Ross 1991; Torres et al. 1994). In general, the food supply is more variable for pelagic species as opposed to benthic species, as seasonal changes are less pronounced in deeper waters. Pelagic species often have a higher and more variable energy density compared to benthic species. This is attributed to the generally more variable food supply for pelagic species as opposed to benthic species, as seasonal changes are less pronounced in deeper waters (Norrbin and Båmstedt 1984).

Predation, seasonality, and subsequent life cycle strategy has influenced the behaviour and distribution of zooplankton and nekton species. This has consequences for the availability of zooplankton and nekton as a food source for predators, for example, prey species have different depth distribution between seasons (Ainley et al. 1991, 2006; Greely et al. 1999; Flores et al. 2014), prey species shift their horizontal distribution depending on growth and retreat of sea ice (Van Franeker 1992; Van Franeker et al. 1997; Flores et al. 2011) or schooling behaviour of prey species changes with food availability, seasons and/or regions which can change the catchability of this prey species for predators (Hamner et al. 1989; Kawaguchi et al. 2010). Therefore, the quality (in terms of energetic value) of available prey may change between seasons, possibly influencing the physiology, distribution and behaviour of predators (Ainley et al. 2015).

Information on the energetic value of prey can be used to predict the behaviour and population dynamics of predators, and to gain insight into key trophic interactions between species (Trathan et al. 2007). It is furthermore important for the calculation of the energy flux through trophic levels of marine ecosystems (Goldsworthy et al. 2001; Lea et al. 2002), the investigation of the importance of a particular prey species in the diet of a predator (Cherel and Ridoux 1992; Lea et al. 2002) and for the use in bioenergetics models (e.g. Hartman and Brandt 1995). The aim of this review is to summarize the knowledge on the energy density of zooplankton and nekton species of the Southern Ocean, for the potential utilization in trophodynamic studies and bioenergetic models. Although the focus is on zooplankton and nekton, benthic species are included. Previously unpublished data are also included in this study.

## Methods

### Southern Ocean environmental framework

South of the STF, the Southern Ocean comprises different water masses and zones with distinct characteristics, separated from each other by several fronts and currents, and is thus not ecologically uniform (Pakhomov and McQuaid 1996; Belkin 2007). Large regions such as the continental shelf and slopes, sub-Antarctic and Antarctic Island groups, features of different fronts, the deep ocean, banks and basins and large gyre systems can be separated having distinct environmental features (Grant et al. 2006). The dominating current of the Southern Ocean is the Antarctic Circumpolar Current (ACC), driven by westerly winds (Orsi et al. 1995; Belkin 2007). The surface water of the ACC has a northern boundary at the Sub-Antarctic Front (SAF). Within the ACC, the Antarctic Polar Front (APF) marks the boundary between warmer sub-Antarctic water and cold Antarctic surface water. The surface waters of the ACC do not show a clear boundary to the south, its properties being rather uniform from the APF to the continental margins. However, in the underlying circumpolar deep water a Southern Boundary (SB) of the ACC occurs (Orsi et al. 1995), which has been found to also influence the physical features of the overlying water (Nicol et al. 2000; Dinniman et al. 2011). The Weddell and Scotia Seas also have different characteristics and they are separated by the Weddell–Scotia confluence separating the ACC from the Weddell Gyre (Orsi et al. 1995; Belkin 2007). Although, the ACC consisting of aforementioned fronts is the classical view based on studies mainly conducted in the Drake Passage, the frontal structure can be more complex in different areas. More details on this can be found in Solokov and Rintoul (2009). Along the margins of the continent there is a westward current, the Antarctic Slope Current. The waters of the continental shelf and the oceanic waters are separated by the Antarctic Slope Front (Jacobs 1991), which in areas where the continental shelf is narrow coincides with the slope current (Heywood et al. 1998). In between the major currents there are various eddies, the largest being the Weddell Gyre and the Ross Gyre (Riffenburgh 2007). Temperature and salinity gradients often coincide with the shelf breaks leading to a separation between coastal and oceanic areas (Ainley and Jacobs 1981; Van de Putte 2008). Broadly, the oceanic area south of the APF can be separated in (from north to south) a permanent open ocean zone, a seasonal ice zone (SIZ) and a coastal and continental shelf zone, which are regarded as different sub-systems with specific mechanisms controlling nutrient and phytoplankton dynamics (Tréguer and Jacques 1992). More information in biogeographic regions can be found in De Broyer and Koubbi (2014).

### Measuring energy density

#### Bomb calorimetry

Bomb calorimetry is the most direct method to analyse the energy content of a species. A bomb calorimeter establishes the energy density (the amount of energy per unit mass) of a plant or animal tissue sample by measuring the heat released when that sample is completely oxidized. The sample is placed in a combustion chamber filled with oxygen, which is surrounded by water. After ignition, the temperature rise in the surrounding water is measured and converted to calorific density. If a sample causes 1000 g of water to rise with 1 °C, the calorific content of the sample is 1 kilocalorie (kcal; Shul’man 1974; Robbins 1983). The calorific density (cal g^−1^ weight) will then depend on the weight of the sample. To determine the whole-body energy density of an animal using bomb calorimetry, the animal is dried and homogenized. After ignition in the bomb calorimeter, the calorific density of the tissue per gram dry weight (DW) is obtained, DW representing the weight of the organic and inorganic contents of the body without any water. Following the Système international d’unités (SI), energetic densities are expressed in joule (J) or kilojoules (kJ). One kilocalorie equals 4.184 kJ.

Depending on the intended use of the data, the energy density can be expressed in several ways. Expression in kJ g^−1^ wet weight (WW) can be useful in studies of trophic relationships and predator distribution/abundance, for instance to translate energetic requirements into food requirements (in number of individuals or kg) and is thus relevant for ecological considerations (Båmstedt 1986; Van Franeker 1992; Flores et al. 2008). However, the wet weight energy content of an individual is strongly related to its water content, the determination of which is a potential source of error. Samples are often weighed after being stored frozen and freezing samples causes dehydration. Calculating the ‘wet’ energetic value can, therefore, be skewed, as a lower water content will result in a higher wet weight energetic value (Hislop et al. 1991). Using fixation solutions also often results in loss of water or lipids and can, therefore, bias the relationship between WW, DW, chemical composition and energy content (Lamprecht 1999). Therefore, expression of energy density in kJ g^−1^ DW can be a better tool for comparison of the energy density within and between species.

As DW includes inorganic material, expression of the energetic density in kJ g^−1^ ash-free dry weight (AFDW), representing the mass of only the organic part of the body or tissue, can in some cases be a more suited unit of measurement, for instance for growth and translocation studies (Lamprecht 1999). For energy comparison between tissues it is also more useful to use AFDW, because different tissues often have different ash contents (Lamprecht 1999). Although the literature sources suggest that ash content can be determined using the residue in the calorimeter cup after combustion (Lamprecht 1999), the more accurate determination is to make an independent estimate of the ash content of an organism (Paine 1971; Craig et al. 1978; Cherel and Ridoux 1992).

Measurements of organisms with high ash content can yield unrealistic energetic values. Ash consisting of high proportions of CaCO_3_ or other decomposable salts can cause endothermic reactions when subjected to the high temperatures present in the bomb calorimeter, leading to a loss of heat within the calorimeter and consequently an underestimation of the energy density (Paine 1964, 1971). This error increases with increasing ash content (Paine 1971). Therefore, caution should be taken with ash contents higher than 25% (Paine 1971). Determination of the proportion of ash can also lead to errors due to the decomposition of salts (Paine 1971).

Measurements of energetic values lower than 17 kJ g^−1^ AFDW (the energetic density of carbohydrates) should be considered with caution, as they may be due to a wrong determination of ash content or to contributions of inorganic reactions during burning (Lamprecht 1999). Even though substances with lower calorific values exist, such as pyruvic acid and glycine, etc., it is unlikely that these substances substantially lower the energetic values of an individual organism (Paine 1971).

A bomb calorimeter typically oxidizes nitrogen to a greater degree than most aquatic organisms (except microorganisms), giving a higher estimate of energy than is actually available to a consumer. To account for this extra energy, a nitrogen correction can be used (Kersting 1972; Salonen et al. 1976). However, for such a correction it is necessary to know the amount of nitrogen in the sample, and correction can possibly vary depending on the organism (Kersting 1972). The energy density values obtained by bomb calorimetry are usually not corrected for nitrogen and may thus be slightly overestimated.

Bomb calorimetry measures the energy content of an organism as a whole. Part of this energy can; however, not be used by the consumer because food is often not completely digested or metabolized. Incomplete catabolism of protein leaves compounds (ammonium, urea, uric acid and creatinine) that are lost in urine (Brody 1945; FAO 2003). The digestibility of chitin, the main component of the exoskeleton of crustacea, can differ between species (Danulat 1987; Jackson et al. 1992), and carbohydrates can have indigestible parts often referred to as dietary fibre (FAO 2003). The energy density determined using bomb calorimetry is thus the gross energy of an organism. This, in contrast to, e.g. metabolizable energy or digestible energy, represents the total amount of energy that is potentially available (Brody 1945; Brett and Groves 1979; FAO 2003). For detailed studies that require knowledge on digestible energy, correction factors and recommendations can be found in Brody (1945) and the FAO (2003). Although analysing fresh tissue is best when using bomb calorimetry, freezing is regarded as the most suitable preservation method for samples, as chemical preservation methods (e.g. ethanol or formaldehyde) significantly affect the results (Giguère et al. 1989; Benedito-Cecilio and Morimoto 2002; Hondolero et al. 2012).

#### Proximate composition

Apart from ash and water fractions, organisms have an organic fraction that can be regarded as being composed of lipids, proteins and carbohydrates. By analysing the relative proportion of these components in the body of an organism, the energetic value can be reconstructed using energetic conversion factors (Paine 1971).

The energy content of the different fractions can show slight variations due to differences in molecular structure (Båmstedt 1986), but conversion factors commonly used are 23.64 kJ g^−1^ AFDW (5.65 kcal g^−1^) for proteins and 16.97 kJ g^−1^ AFDW (4.1 kcal g^−1^) for carbohydrates (Brett and Groves 1979). For lipids, an energy content of 39.54 kJ g^−1^ AFDW (9.45 kcal g^−1^) has often been used (Paine 1971 and references therein; Brett and Groves 1979). These values represent gross energy content of the compounds (Brody 1945; Brett and Groves 1979), which, similar to bomb calorimetry, does not take into account potential differences in digestibility between animals and substrates, and lost protein compounds (Brody 1945; FAO 2003). A factor of 36.40 kJ g^−1^ AFDW (8.7 kcal g^−1^) is suggested to be more appropriate for lipids, because lipid content in the body may be overestimated due to impurities in the lipid extract (Craig 1977; Craig et al. 1978). This may, however, vary between methods used (FAO 2003). As the energy density of lipids is almost twice as high as that of protein, higher lipid contents often result in a higher energetic value (Anthony et al. 2000). Therefore, differences in the lipid content of organisms can often predict differences in energy density. There are exceptions to this rule, however, as the energy density can also change significantly due to changes in, e.g. water or protein content, particularly during growth (Shul’man 1974; Donnelly et al. 1994). In addition, changes in protein content cause greater changes in an organism’s weight compared to lipids (Shul’man 1974).

As carbohydrates usually contribute very little to the total dry body composition, this constituent is sometimes not considered in proximate analysis (Craig et al. 1978). The protein content of a body is sometimes estimated by measuring the total nitrogen content of a sample and then multiplying this with a factor 6.25, which is known as the Kjeldahl method (Craig et al. 1978). The protein content estimated using this method is often referred to as crude protein. For the energetic contribution of chitin to the total energy density, the same conversion factor as for carbohydrate is usually used (Clarke 1980; Donnelly et al. 1994). Such factors cannot always accurately represent the potentially large variability of energy content of proximate compounds. Therefore, estimating the energetic content by means of proximate compositions is potentially subject to more error than bomb calorimetry (Henken et al. 1986; Kamler 1992; Hartman and Brandt 1995; Higgs et al. 1995).

Several studies found a good agreement between energy densities estimated using proximate composition and measured with bomb calorimetry (Paine 1971; Vollenweider et al. 2011). Other studies, however, found significant discrepancies between energy densities established using both proximate composition and bomb calorimetry (Craig et al. 1978; Henken et al. 1986; Kamler 1992). Energetic densities based on proximate composition were on average 4.4% higher than values obtained with bomb calorimetry in Craig et al. (1978), while they were on average 3–4% lower in Henken et al. (1986). The conversion factors do not take into account potential differences in heat of combustion of protein, depending on their amino acid composition, or the contribution of dietary fibre to carbohydrates, which have a lower energetic density (FAO 2003). Furthermore, methods used for measuring the relative contribution of different proximate compounds, as well as calculation of the energetic value, often differ between studies (Henken et al. 1986). Therefore, bomb calorimetry is considered the preferable method for energy density estimation (Henken et al. 1986; Kamler 1992; Hartman and Brandt 1995; Higgs et al. 1995). An advantage of proximate composition measurements is that changes in energy density can be related to changes in particular components that can give additional information on, e.g. ecological strategies, feeding activity, trophodynamics and reproductive status (Lawrence and Guille 1982; McClintock and Pearse 1987; Donnelly et al. 1994). A clear recommendation on the preservation of samples for proximate composition analysis was not found, but samples are usually processed directly or stored frozen.

#### Water content, carbon content and energy density

A relationship between energy density and water content is often found, showing an increase in water content with decreasing energy content (on a WW basis) and vice versa (Båmstedt 1981; Torres et al. 1994; Hartman and Brandt 1995). This can be attributed to water and lipids or protein replacing each other, depending on age, season and reproductive state (Torres et al. 1994; Hartman and Brandt, 1995; Lea et al. 2002; Tierney et al. 2002; Van de Putte et al. 2006). For example, the water content increases when lipids (or protein) are combusted (Torres et al. 1994). The relationship between water, lipid and protein content in fish changes with age because younger individuals would use the protein to build-up the body, but when growth ceases and protein metabolism stabilizes, the fish switch to the accumulation of fat (Shul’man 1974). Protein growth occurs in adult fishes in the form of gonad development (Shul’man 1974). Protein and lipid accumulation can, however, also depend on availability and composition of food. For example, in two species of anchovy with similar energy densities, one species had less available food, was larger at same age and contained more protein and less fat, while the other species had more food available, was fatter, but also smaller and contained less protein (Shul’man 1974). The water content/energy density (WW) relationship is also common in crustaceans (Torres et al. 1994). Exceptions are found, however, in for instance decapod, amphipod and krill species, where water and lipids do not replace each other, but increase or decrease simultaneously, or where changes in one of the fractions do not lead to changes in the other (Torres et al. 1994).

Relationships have also been found between total carbon content and energy density. Platt and Irwin (1973), Salonen et al. (1976), Finlay and Uhlig (1981), Gnaiger and Bitterlich (1984) and Normant et al. (2002) show regressions to calculate energy density. Different studies show relationships using different parameters and variable methods to establish both carbon content and energy density, making it hard to compare them. Measurements were done on phytoplankton (Platt and Irwin 1973), protozoa (Salonen et al. 1976; Finlay and Uhlig 1981) and crabs (Normant et al. 2002). Platt and Irwin (1973) make a regression calculating calories mg^−1^ DW using the total % carbon, while Salonen et al. (1976) calculate kJ g^−1^ AFDW using the total % carbon, the former having a negative intercept, while the latter has a positive one. The relationship found by Normant et al. (2002), between kJ g^−1^ DW and % carbon, also has a negative intercept, and a relatively low *R*^2^ (0.61), suggesting that a relatively low proportion of the variability was explained by the regression. Finlay and Uhlig (1981) calculate energy density in terms of kJ g^−1^ DW based on mg C mg^−1^ DW. Färber-Lorda et al. (2009a) shows a regression between carbon and energy in krill, with values based on mg ind^−1^ and J ind^−1^. In addition to regressions, factors to convert carbon to energy density were suggested. Salonen et al. (1976) suggested a conversion factor of 45.7 kJ (AFDW) g^−1^ organic carbon while Finlay and Uhlig (1981) suggested 46 kJ g^−1^ organic carbon. A conversion factor of 50.2 kJ g^−1^ C was suggested based on measurements on the amphipod *Themisto compressa*, caught in the North Atlantic (Williams and Robins 1979). Due to differences in regression slopes and intercepts, measured species or species groups, and differences in units used, it remains unclear if the conversion factors and regressions can be used in a general context. It is also likely that season, region, organism, size and age will affect the carbon–energy density relationship, and these influences need to be assessed. Therefore, carbon content was not used in this review to estimate the energy density of species.

### Data and statistics

In this review, we aimed to express all energy density values in kJ g^−1^ DW for species comparison and in kJ g^−1^ WW for use in ecological studies. When possible, the energy density values obtained from the literature were recalculated to kJ g^−1^ DW and/or kJ g^−1^ WW using given energy densities, species weights or water contents reported in the references concerned. Energy density values, determined by proximate composition, were calculated by the original authors using a factor of 36.40 kJ g^−1^ AFDW for the conversion of the lipid fraction, unless stated otherwise. Protein values represent actual measurements derived from true protein content analysis. When crude protein measurements were used in the original paper, this is specified. We also calculated energy densities from references reporting only proximate composition values (usually given in %WW) using the above mentioned conversion factors. When the carbohydrate fraction was not given in the source, we assumed it to be the remainder of 100% minus the other fractions (water content, lipids, carbohydrate, protein, ash, and where relevant, chitin). The lengths of fish reported in this review are given in standard length (SL), measured from the most forward part of the head to the end of the vertebrae. Some lengths are given in total length (TL), which is measured from the most forward part of the head to the end of the caudal fin.

Previously unpublished data obtained during two expeditions have been included in this review. Individual zooplankton and nekton species were collected on board the RV Polarstern in the Weddell Sea (PS81: August–October 2013) and in the Lazarev Sea (PS89: December/January 2014/2015), using Rectangular Midwater Trawls (RMT) and Surface and Under-Ice Trawls (SUIT). Details on sampling procedures, research area and environmental conditions for PS81 and PS89 can be found in Schaafsma et al. (2016) and Flores et al. (2015), respectively. After collection, zooplankton and nekton species were frozen at − 20 °C. Before the analysis of energetic value, samples were defrosted, blotted dry, and length and WW were measured. Then samples were freeze-dried until complete desiccation and re-weighted to determine DW and water content. After homogenization, a subsample of approximately 0.5 g was used for calorimetry. If necessary, individuals were pooled to obtain a sufficient amount of material to enable energy density measurements. The energy density (in kJ g^−1^ DW) of samples was determined with an isoperibol bomb calorimeter (IKA C2000 basic), calibrated with benzoic acid. Benzoic acid (29.62 kJ g^−1^ DW) was added to samples that were too small to obtain a minimum sample weight of 0.5 g. Some jelly fish body parts did not combust in the bomb calorimeter, most likely due to high ash contents (> 75%DW). These tissues were then measured again using a sample consisting of half tissue, half benzoic acid. The AFDW of the jelly fish was obtained by drying a homogenized sample to constant mass at 60 °C, followed by 6 h incineration at 500 °C.

In datasets with a sufficient sample size, energy densities were compared using two-way ANOVA followed by a non-parametric Tukey’s HSD post hoc test. Linear relationships between DW and energy content were established using ln-transformed data (Van de Putte et al. 2006). Linear relationships between water content and wet weight energy density were also investigated. Slopes and intercept of regression models were compared using ANCOVA (Hartman and Brandt 1995). All analyses were performed with R version 3.3.1 (R Core Team 2015). Seasons listed within the tables are defined as stated by the authors, or as summer for December to February, autumn for March to May, winter for June to August and spring for September to November. It should be kept in mind that environmental conditions may vary within a month depending on region. All data used in this review, including the previously unpublished data, are available as part of the SCAR Southern Ocean Diet and Energetics Database, which is a compilation of diet and energetics data from Southern Ocean studies. More information on use and contributing can be found at https://www.scar.org/data-products/southern-ocean-diet-energetics/.

## Energy density of zooplankton and nekton species

### General overview

**Fig. 1:**
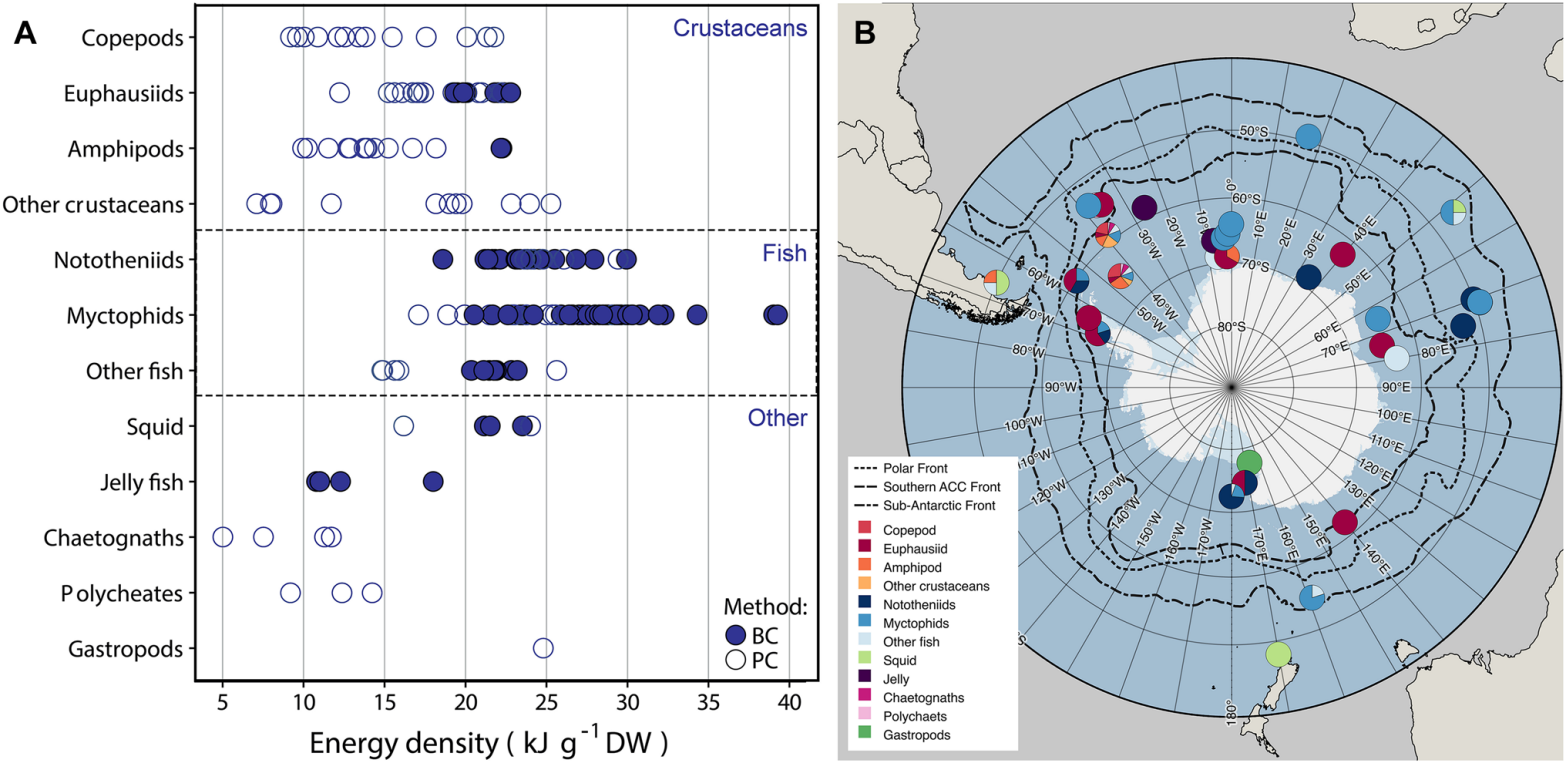
**a** Overview of energy density records per species group. One point represents an average energetic value per species and per record. A distinction is made between measurements done using bomb calorimetry (BC) and proximate composition (PC). Note that one literature source can contain multiple energy density records, for instance of different species or developmental stages, and that, therefore, one point does not represent one literature source. **b** Overview map of energy density records, including several fronts. One point on the map represents one source. Therefore, a single point can include multiple measurements on a single species or measurements of multiple species from a single group. Approximate locations were derived from the source material. The map was made using Quantarctica from the Norwegian Polar Institute (Matsuoka et al. 2018). Mean front positions were taken from Solokov and Rintoul (2009). Previously unpublished data are included

Energetic densities of zooplankton and nekton species from sub-Antarctic and Antarctic waters collected and found in the literature included crustaceans such as copepods, euphausiids, amphipods, mysids and decapods, fish, squid, and gelatinous species. The numbers of records varied greatly between groups and species. Some species have been given more attention than others which is often related to their abundance, importance in the diet of top predators, commercial interest and catchability. Figure 1 shows an overview of all reported dry weight energy densities per species group and the locations at which recorded animals were sampled.

The majority of measurements of energy content in Antarctic crustaceans were conducted on euphausiids. The most comprehensive studies of energy density of crustaceans other than euphausiids were conducted by Donnelly et al. (1994) and Torres et al. (1994), using proximate composition. These studies provide, to our knowledge, almost the only records of energy densities of copepod, amphipod, decapod, mysid and ostracod species, which were caught in autumn and winter in the north-western Weddell Sea and the southern Scotia Sea. Donnelly et al. (1994) noted that their estimates of energy density are in general relatively low due to the incomplete recovery of organic material during analysis. Copepods showed a wide range of dry weight energy density values including very low values. Other low values were in general found for amphipods and ostracods. Amphipods have the highest skeletal ash, suggesting a more robust exoskeleton compared to copepods, euphausiids, decapods and mysids (Percy and Fife 1981; Torres et al. 1994). This can result in a lower dry-weight energy density because smaller proportion of the DW encompasses organic material. Amphipods furthermore have the highest chitin content (Donnelly et al. 1994; Torres et al. 1994). However, two measurements on amphipods using bomb calorimetry yielded an energy density similar to the other crustaceans. It is unclear if this was is an artefact of the different methods used, as all other energy densities were estimated using proximate composition, or due to a different life cycle and/or distribution of the species. Ostracods had a low lipid content and slightly higher ash content compared to other crustaceans except amphipods (Donnelly et al. 1994).

In terms of energetic measurements, fish are the most studied organisms in the Southern Ocean. The main focus lies on nototheniid, myctophid and bathylagid species. The lipid content of myctophids is in general high, while nototheniids are more variable in composition, which shows a difference between the two families that is possibly related to habitat use (Lenky et al. 2012). This is reflected in their dry-weight energy density, which was generally high for myctophids, while for nototheniids it ranged from values similar to crustaceans to values similar to myctophid fish. Dry-weight energetic densities of fish from other families, including *Bathylagus antarcticus* and *Notolepis coatsi*, were also comparable to those of crustaceans or the lower end of the range of nototheniids (Fig. 1). A similar range was found for squid.

Dry-weight energy densities of other groups showed relatively low values with the exception of a gastropod species, *Clione limacina antarctica* (Bryan et al. 1995). Measuring the energy content of gelatinous species is difficult due to their low proportion of organic material (high ash content), and high water content. A large part of the inorganic ash can be attributed to salt; a result from the large volume of sea water constituting the bulk of the organism’s tissue (Percy and Fife 1981; Norrbin and Båmstedt 1984). In jellyfish it is thought that residual water remains, even after drying to constant mass. This residual water is estimated to be 11.7% DW (Larson 1986; Doyle et al. 2007). For these reasons bomb calorific measurements and proximate composition estimates of gelatinous species should be considered with caution (Doyle et al. 2007). The high ash content can furthermore explain the low dry-weight energy density values of gelatinous species such as jelly fish, salps and siphonophores.

### Crustaceans

#### Copepods

Copepods are the numerically dominant zooplankton group and often also dominate in biomass (Foxton 1956; Schnack-Schiel et al. 2001; Atkinson et al. 2012; David et al. 2017). Therefore, they are an important part of the diet of many zooplanktons, fish and some top predator species (Laws 1977; Gon and Heemstra 1990; Hubold and Ekau 1990; Bocher et al. 2002; Van Franeker et al. 2002). Many species found in the Antarctic and sub-Antarctic regions have a wide distribution and are found north of the STF, sometimes even as far north as the Arctic Ocean (Kouwenberg et al. 2014). Of the total 388 species that have been reported to occur in the Southern Ocean, 53 are endemic south of the APF (Kouwenberg et al. 2014) and often rare. Many copepods can also be found residing within the sea ice (Schnack-Schiel et al. 2001; Arndt and Swadling 2006).

The energy densities of copepods estimated in Donnelly et al. (1994) ranged between 9.0 and 21.8 kJ g^−1^ DW. Highest energy densities were from *Paraeuchaeta antarctica* (21.8 kJ g^−1^ DW), *Calanus propinquus* (21.3 kJ g^−1^ DW) and *Calanoides acutus* (17.6 kJ g^−1^ DW) which were all caught in autumn. All three species have a wide distribution and occur from south of the STF to the Antarctic continent (Kouwenberg et al. 2014). The other species analysed in Donnelly et al. (1994) showed energy densities below 13.8 kJ g^−1^ DW. An overview of recorded copepod average energy density measurements including, where possible, values expressed in kJ g^−1^ WW can be found in Table S1 of the electronic supplement.

Some observations on energy content of copepods by Donnelly et al. (1994) can be explained by their life cycle, overwintering strategy and/or food. Species such as *C. acutus* and *C. propinquus* are mainly herbivorous and have high lipid levels (Donnelly et al. 1994), resulting in a relatively high energy density. More omnivorous species, such as *Euchirella rostromagna* and *Gaetanus tenuispinus*, or carnivorous species, such as *Heterorhabdus* spp. have lower lipid levels (Donnelly et al. 1994). There are, however, exceptions to this pattern: the carnivorous *Paraeuchaeta antarctica* was found to have a high lipid content and the herbivorous *Rhincalanus gigas* has a relatively moderate lipid content, the latter attributed to their more flexible 2-year life cycle including a delayed reproduction (Donnelly et al. 1994). *Heterorhabdus austrinus* continues to feed during winter which is reflected in higher protein content and lower lipid content compared to its congener *H. farrani*, which does not feed during winter. Their estimated energy content was, however, similar (12.1 kJ g^−1^ DW; Donnelly et al. 1994).

All species that were analysed in two seasons showed a similar or lower energy density in winter compared to autumn, except for *Rhincalanus gigas*. *Calanoides acutus* overwinters at depth in diapause and did not show a difference in proximate composition between seasons which could be attributed to its reduced metabolic rates (Donnelly et al. 1994). This could also be the case for *R. gigas*, although this species has also been found to feed and reproduce during winter (Atkinson 1998). *Calanus propinquus*, overwintering using a combination of continuous feeding, reduction in body integrity and combustion of energy reserves, shows an increase in water level, and a decrease in chitin content and lipid levels from autumn to winter (Donnelly et al. 1994). As *C. propinquus* relies on energy reserves, their energy content can be expected to show large variations between seasons. Changes from autumn to winter were observed in the composition of *Paraeuchaeta antarctica* which was suggested to be a consequence of reproductive demand. Their energy content was, however, similar in both seasons (Donnelly et al. 1994). Studies on the lipids of copepods indicated that seasonal as well as regional variability of lipid content can be found within species, due to differences in food availability, type of food and overwintering strategy (Hagen et al. 1993; Donnelly et al. 1994).

#### Euphausiids

Euphausiids are a major component of Southern Ocean ecosystems. The three most studied species of Euphausiacea are *Euphausia superba*, *Thysanoessa macrura* and *Euphausia crystallorophias*. *Euphausia superba* has a circumpolar distribution, from south of the polar front to the continental shelf, with a majority of the total stock found in the regions of the Antarctic Peninsula and the Scotia Arc (Atkinson et al. 2008; Pakhomov et al. 2000; Flores et al. 2012). *Thysanoessa macrura* has a similar distribution but can also be found north of the SAF (Pakhomov et al. 2000; Atkinson et al. 2012; Flores et al. 2012; Cuzin-Roudy et al. 2014). The distribution and density of *E. superba* has been related to sea ice, although this association differs between seasons, while the smaller *T. macrura* can be found in ice-covered waters but is less ice-associated and often occupies a deeper stratum (Nordhausen 1994; Flores et al. 2012; Haraldsson and Siegel 2014). *Euphausia crystallorophias* is neritic and found close to the Antarctic continent (Nordhausen 1994; Pakhomov and Perissinotto 1996), where they reside in ice-covered waters year-round. For all krill species, larvae, juveniles and adult have different physiological, metabolic and functional adaptations and can, therefore, have different habitat requirements (Cuzin-Roudy et al. 2014). The largest species, *E. superba*, is the most heavily studied due to its high total biomass, its importance in the diet of many top predators and because it is a target species of a growing fishery (Atkinson et al. 2012).

The lowest average energetic density for *E. superba* was 15.2 kJ g^−1^ DW for adults during autumn, estimated using proximate composition (Torres et al. 1994). The highest density found in the literature is 22.7 kJ g^−1^ DW of gravid females at South Georgia during summer (Clarke 1980), although another source reports a somewhat lower energetic density for gravid females (20.1 kJ g^−1^ DW) found at Elephant Island (Ishii et al. 2007). Both aforementioned energy densities were estimated using proximate composition, but differences in methodological details used could have resulted in different values. Ishii et al. (2007), for instance, did not take the chitin fraction into account and details on the methods used for different components are undescribed. For the energy densities of *T. macrura*, *E. crystallorophias* and *Euphausia frigida*, estimates using bomb calorimetry, proximate composition and calculations using published equations (Färber-Lorda 1986; Torres et al. 1994; Ainley et al. 2003; Ruck et al. 2014) suggest that the energy density of these krill species is similar to that of *E. superba.* Bomb calorific measurements on adult and juvenile *T. macrura* from the southern Indian Ocean showed that individuals at one station (6.1 and 5.4 kJ g^−1^ WW, respectively) had higher WW energy density values than individuals from another station (5.5 and 4.8 kJ g^−1^ WW, respectively; Färber-Lorda 1986). A measurement of the mesopelagic, circumpolarly distributed *Euphausia triacantha* (Piatkowski 1985; Atkinson et al. 2012) showed that this species had a relative low energy density compared to the other euphausiid species from the same study (Torres et al. 1994). An overview of recorded euphausiid average energy density measurements including, where possible, values expressed in kJ g^−1^ WW can be found in Table 1.

**Table 1:** Overview of the average energy density of several euphausiid species ±, were available, the standard error (SE) or standard deviation (SD) as given in the original source

Season	Location	*n*	Stage	Water	Mean energy density		Method	Source
				Content (%)	kJ g^−1^ WW	kJ g^−1^ DW		
*Euphausia superba*								
Summer	South Georgia	5–20	Female (gravid)	76.0	5.45^a,b^	*22.66*	PC	Clarke (1980)
	Elephant Island	4	Female (gravid)	75.9 ± 0.4 SE	4.80^a,c^ ± 0.05 SE	*20.08*	PC	Ishii et al. (2007)
	Southern Indian Ocean	7	Female (spent)		4.88 ± 0.78 SD		MBC	Färber-Lorda et al. (2009a)
	Lazarev Sea	3 (p)	Female	73.8 ± 1.9 SD	5.54 ± 0.73 SD	22.27 ± 0.72 SD	BC	This study (PS89)
	Southern Indian Ocean	15	Female		6.31 ± 0.88 SD		MBC	Färber-Lorda et al. (2009a)
	WAP	(p)	Female			22.0 ± 0.3 SE	BC	Ruck et al. (2014)
	Elephant Island	2	Female	77.7 ± 1.3 SE	4.16^a,c^ ± 0.33 SE	*17.41*	PC	Ishii et al. (2007)
	South Georgia	5–20	Male	80.1	3.83^a,b^	*19.22*	PC	Clarke (1980)
	Southern Indian Ocean	10	Male		4.76 ± 0.96 SD		MBC	Färber-Lorda et al. (2009a)
	WAP	(p)	Male			19.5 ± 0.5 SE	BC	Ruck et al. (2014)
	Elephant Island	4	Male	78.9 ± 0.5 SE	3.73^a,c^ ± 0.12 SE	*15.61*	PC	Ishii et al. (2007)
	Elephant Island	2	Male (sub-adult)	77.9 ± 0.3 SE	4.09^a,c^ ± 0.03 SE	*17.11*	PC	Ishii et al. (2007)
	Lazarev Sea	2 (p)	Juvenile	75.1 ± 3.5 SD	5.63 ± 1.19 SD	22.38 ± 0.44 SD	BC	This study (PS89)
	Southern Indian Ocean	10	Juvenile		5.59 ± 0.76 SD		MBC	Färber-Lorda et al. (2009a)
	WAP	(p)	Juvenile			20.8 ± 1.7 SE	Calc	Ruck et al. (2014)
	Elephant Island	1	Juvenile	78.3	4.0^a,c^	*16.74*	PC	Ishii et al. (2007)
	WAP	9		77.0 ± 2.7 SD	*5.01*	21.8 ± 0.7 SD	BC	Nagy and Obst (1992)
				75.7	4.86	*20.0*	PC	Yanagimoto et al. (1979)^e^
Summer/autumn	East Antarctica	1			4.47		BC	Tamura and Konishi (2009)
Autumn				75	5.31	*22.22*	PC	Márquez et al. (1978)^e^
	Weddell Sea	23	Adult	73.3 ± 3.4 SD	4.07^f^	*15.24*	PC	Torres et al. (1994)
				*76.5*	*4.71*	20.0	BC	Jackson (1986)
Winter	Scotia Sea	32	Adult	77.3 ± 3.4 SD	3.80^f^	*16.75*	PC	Torres et al. (1994)
*Thysanoessa macrura*								
Summer	WAP	(p)				28.5 ± 2.8 SE	Calc	Ruck et al. (2014)
	Southern Indian Ocean	1 (p)	Adult		5.52		MBC	Färber-Lorda (1986)
	Southern Indian Ocean	1 (p)	Adult		6.12		MBC	Färber-Lorda (1986)
	Southern Indian Ocean	1 (p)	Juvenile		4.76		MBC	Färber-Lorda (1986)
	Southern Indian Ocean	1 (p)	Juvenile		5.35		MBC	Färber-Lorda (1986)
	Southern Indian Ocean			*74.2*	*5.42*	*21.00*	PC	Färber-Lorda et al. (2009b)
Autumn	Weddell Sea	1 (p)		70.4	5.04^f^	*17.02*	PC	Torres et al. (1994)
Winter	Scotia Sea	6 (p)		76.9 ± 1.2 SD	3.72^f^	*16.10*	PC	Torres et al. (1994)
*Euphausia crystallorophias*								
Summer	Ross Sea	4 (?)	Adult			*19.33*	BC	Ainley et al. (2003)
	WAP	(p)				21.8 ± 0.8 SE	Calc	Ruck et al. (2014)
Autumn				80.6	3.85	*19.85*	BC	Green and Gales (1990)
				71.7	6.45^d^	*22.79*	BC	Green and Gales (1990)
*Euphausia triacantha*								
Winter	Scotia Sea	9 (p)		76.1 ± 3.6 SD	2.92^f^	*12.22*	PC	Torres et al. (1994)
*Euphausia frigida*								
Summer	Southern Indian Ocean	1 (p)			4.62		MBC	Färber-Lorda (1986)

Method used for energy density estimates are bomb calorimetry (BC), micro-bomb calorimetry (MBC), proximate composition (PC) and are calculated using published equations from Färber-Lorda et al. (2009a; Calc). Energy densities given in italics represent values that were converted using information from the given sources. *n* represents the number of samples measured. Where this expresses samples of pooled individuals, this is indicated with (p)

^a^Energy density calculated with an energetic value of 39.54 kJ g^−1^ AFDW (9.45 kcal g^−1^) for lipids

^b^A factor of 4.1864 was used to convert calories to joules

^c^Energy density calculated excluding chitin

^d^Sample taken from bird stomach contents, in which the energetic value is potentially overestimated due to water removal in stomach

^e^From Barrera-Oro (2002)

^f^A factor of 4.19 was used to convert calories to joules

The energy density of *E. superba* varies between regions, seasons, sexes and states of sexual maturity. Mature females have a high energy density and lose up to 55–58% of their lipids when spawning, resulting in a lower energetic value (Clarke 1980; Färber-Lorda et al. 2009b). *Euphausia superba* spawns from December to April with a peak in January (Ross and Quetin 1986; Pakhomov 1995; Spinidonov 1995). During summer, the energetic density of males is relatively low compared to juveniles and females (Clarke 1980; Färber-Lorda et al. 2009a). Studies suggest that this is due to differences in lipid accumulation, which was found to be low in males and at a maximum in maturing females, although a lot of variance was found (Pond et al. 1995; Mayzaud et al. 1998; Färber-Lorda et al. 2009a; Ruck et al. 2014). Lower lipid content in males is assumed to be a result of a higher investment of energy in growth to increase reproductive success (Ruck et al. 2014). Virtue et al. (1996) suggested that low accumulation of lipids in male krill is a result of a higher sexual activity. Multiple linear regressions between dry weight, carbon content, and lipid content versus energy content of *E. superba*, reported as values individual^−1^, can be found in Färber-Lorda et al. (2009a).

Similar differences in lipid content between males and females were found for *T. macrura* (Färber-Lorda and Mayzaud 2010).The lipid content of *E. superba* and *T. macrura* showed a high local variability in several studies (Pond et al. 1995; Hagen et al. 1996; Mayzaud et al. 1998; Färber-Lorda et al. 2009a; Färber-Lorda and Mayzaud 2010; Ruck et al. 2014; Kohlbach et al. 2017). In *E. superba* lipid, but also protein content, was found to be highly variable within a single population during several seasons, and the variety within a season can be greater than between seasons (Torres et al. 1994; Mayzaud et al. 1998; Ruck et al. 2014). This intra-seasonal variation can be attributed to a patchy and/or regionally variable distribution of available food (Mayzaud et al. 1998; Ruck et al. 2014; Virtue et al. 2016; Schaafsma et al. 2017).

As the spawning seasons of *T. macrura* and *E crystallorophias* are somewhat earlier in the year compared to *E. superba*, differences in timing of the peak energetic value can be expected between species. The spawning season for *T. macrura* ranges from June to January with a peak from September to November (Haraldsson and Siegel 2014), while *E. crystallorophias* spawn in November/December (Pakhomov and Perissinotto 1996; Falk-Petersen et al. 2000). Both species use energy reserves accumulated in summer and autumn to overwinter and reproduce, which ensures that their larvae can feed on the spring phytoplankton blooms (Falk-Petersen et al. 2000; Vallet et al. 2011). *Euphausia superba* needs the spring and summer phytoplankton blooms for sexual maturations, mating and egg development (Cuzin-Roudy et al. 1999). Due to the lack of data, however, these differences in life cycles do not become clear in a seasonal variability of their energetic density. Regarding lipid contents, *E. crystallorophias* showed steady decrease of lipid content over winter and the following spawning period in spring. Lipid content increased again in late spring/summer which was found to coincide with elevated chlorophyll *a* content in the water column (Clarke 1984). Larger sized individuals of *E. triacantha* showed a higher lipid level and lower water content than smaller sized individuals. Seasonal changes in composition suggests that this species combusts tissue during winter (Torres et al. 1994).

#### Amphipods

The 820 amphipod species recorded in the Southern Ocean occupy a very wide variety of ecological niches and have a large range of feeding strategies (Dauby et al. 2001; De Broyer et al. 2001; Dauby et al. 2003; Zeidler and De Broyer 2014). The amphipods can be divided in gammarid and hyperiid amphipods. The gammarid amphipods are mainly benthic with few pelagic species. Some gammarids, such as species from the genus *Eusirus*, have been found closely related to the sea-ice underside (Flores et al. 2011; David et al. 2017). The hyperiid amphipods are mainly pelagic and have been found to be important prey species for top predators such as several bird species (Ridoux 1994; Bocher et al. 2001). The swarming *Themisto gaudichaudii* occurs in high abundances in the sub-Antarctic and Antarctic regions (Kane 1966).

The energy density of several amphipod species from the Weddell and Scotia Seas was estimated using proximate composition by Torres et al. (1994). The lowest value of 9.9 kJ g^−1^ DW, was from the gammarid amphipod *Parandania boecki* collected in winter (Table S2). This species also had the highest water content and is the deepest living. It has furthermore been found to have low lipid levels and to be feeding on coelenterates (Reinhardt and Van Vleet 1986). The highest energetic density of 18.2 kJ g^−1^ DW, was from the hyperiid amphipod *Cyllopus lucasii* collected in autumn (Torres et al. 1994). The relatively high energy density expressed in kJ g^−1^ WW is a result of the water content of 68.7% (of WW), which is relatively low compared to that of other amphipods or euphausiids.

Both *C. lucasii* and *Primno macropa* showed a significant decline in energy density in winter compared to autumn (Torres et al. 1994). This could be a result of reproductive activity, but considering what is known about the timing of reproduction, most likely a result of lipid combustion. This was supported by an increase in water content with decreasing lipid content. *Cyllopus lucasii* furthermore showed significant variability in lipid content between regions (Torres et al. 1994). *Themisto gaudichaudii* had a very low energy density of 12.7 kJ g^−1^ DW during wintertime. It was suggested to be a result of reproductive activity, as their reproduction peak is in spring. Mayzaud and Boutoute (2015) found that *T. gaudichaudii* (females), which continues to feed carnivorously over winter, had a relatively stable lipid content year-round. A bomb calorimetry measurement of *T. gaudichaudii* yielded an average energy density of 22.1 kJ g^−1^ DW (Ciancio et al. 2007). Torres et al. (1994) suggested a mixed overwintering strategy for all examined hyperiid amphipods. The gammarid amphipods examined in Torres et al. (1994) are all deeper living species and a business-as-usual overwintering strategy was suggested.

An energy density of 22.3 kJ g^−1^ DW was found for the gammarid *Eusirus microps* during summer in the Lazarev Sea (PS89). *Eusirus microps* has been found in the surface of both open and ice-covered waters during summer (Flores et al. 2011) and winter (Flores et al. 2011; David et al. 2017). All energy density values of amphipods are listed in Table S2 of the electronic supplement.

#### Other crustacea

Energy density values of crustaceans of the orders Decapoda, Mysida and the class Ostracoda were also found in Donnelly et al. (1994) and Torres et al. (1994). Their energy densities, estimated using proximate composition, ranged from 19.0 to 25.3, 18.2 to 24.0, and 7.1 to 11.7 kJ g^−1^ DW, respectively. The decapod *Pasiphaea scotiae* had a higher energy density in autumn compared to winter, while the opposite was found for the decapod *Petalidium foliacium*. The species from Torres et al. (1994) are all deeper living animals, although ostracods have also been found in the under-ice surface (David et al. 2017). Recorded energy density measurements including, where possible, values expressed in kJ g^−1^ WW are listed in Table S3 of the electronic supplement.

### Fishes

In general, there is a strong distinction between coastal and oceanic fish assemblages (Hubold 1991; Kock 1992). The families Myctophidae, Bathylagidae, Gonostomatidae and Paralepidae dominate the fish community of the Southern Ocean’s oceanic waters (Kock 1992; Flores et al. 2008; Duhamel et al. 2014). The oceanic myctophids, or lanternfishes, dominate the meso- and bathypelagic zones in term of species richness, abundance and biomass (references in Duhamel et al. 2014). The cold waters of the Antarctic continental shelf and slope are dominated by the Nototheniidae (Eastman and Eakin 2000; Van de Putte 2008), which are mainly benthic or bentho-pelagic (La Mesa et al. 2004). Other families significantly contributing to the Southern Ocean fish fauna are the Liparidae, Zoarcidae and Macrouridae (Duhamel et al. 2014). The neritic species composition differs between the continental areas, SIZ and around the (sub-)Antarctic islands (Kock 1992). In some species, the larval stages have a different (vertical) distribution pattern than adult individuals of the same species (e.g. Hubold 1990).

The availability of previously unpublished data and data of individual fish kindly provided by colleague researchers, allows for a more detailed description and analysis of the energetic density of the nototheniid *Pleuragramma antarctica*, the myctophids *Electrona antarctica*, *Gymnoscopelus braueri* and the bathylagiid *Bathylagus antarcticus*.

#### *Pleuragramma antarctica*

The notothenoid *Pleuragramma antarctica* is the most abundant pelagic fish in the high Antarctic coastal regions, with an extended range to the South Shetland and South Orkney Islands (Eastman and Hubold 1999; La Mesa et al. 2004; Donnelly and Torres 2008; Van de Putte 2008). It is an important prey species for many fish species and (Eastman 1985) and top predators, including flying birds (Van Franeker et al. 2001), seals (Southwell et al. 2012 and references therein) and penguins (Ainley et al. 1998; Cherel and Kooyman 1998),

Reported and measured average energy density values of *Pleuragramma antarctica* ranged from 21.7 to 27.9 kJ g^−1^ DW (both summer Ross Sea). In East Antarctica, the energy density increased with age, from 21.8 to 25.5 kJ g^−1^ DW in small (52–95 mm) and large, adult (> 105 mm) individuals, respectively (Van de Putte et al. 2010). The water content showed an opposite trend and was higher in the younger group (87.9%) compared to the older one (70.2%; Van de Putte et al. 2010). The energy density of juvenile fish showed a lot of variation, possibly attributed to variability in foraging success (Van de Putte et al. 2010). Therefore, despite differences between size classes, there was no (linear) relationship between size and energy density within the small group. An overview of recorded average energy density measurements of *Pleuragramma antarctica* including, where possible, values expressed in kJ g^−1^ WW can be found in Table 2.

**Table 2:** Average energy densities of *Pleuragramma antarctica*, measured using bomb calorimetry (BC)

Season	Location	*n*	Mean size (mm)	WW (g)	DW (g)	Water content (%)	Mean energy density		Method	Source
							kJ g^−1^ WW	kJ g^−1^ DW		
Summer	Ross Sea	(p)	134^a^ ± 21 SE	23.7 ± 15.2 SE	*4.2*	82.1	5.00	*27.93*	BC	Lenky et al. (2012)
	Ross Sea		70–120					21.76	BC	Ainley et al. (2003)
	WAP		89.9 ± 4.3 SE					24.6 ± 0.4 SE	BC	Ruck et al. (2014)
Autumn	East Antarctica	14	52–95	1.6 ± 0.6 SD	0.2 ± 0.1 SD	87.9 ± 1.1 SD	2.64 ± 0.25 SD	21.83 ± 0.44 SD	BC	Van de Putte et al. (2010)
	East Antarctica	2	> 105	6.1 ± 0.1 SD	1.8 ± 0.04 SD	70.2 ± 2.8 SD	7.59 ± 0.65 SD	25.52 ± 1.18 SD	BC	Van de Putte et al. (2010)

Numbers in italics represent values that were converted using information from the given sources. Sizes are in standard length. *n* represents the number of samples measured. Where this expresses samples of pooled individuals, this is indicated with (p). Where available, the standard error or standard deviation as given in the original source is added (±)

^a^Measured in total length (TL)

The relatively low energy density of young *Pleuragramma antarctica* could possibly be due to their small size. The energy density of adult *Pleuragramma antarctica* is closer to that of the myctophid fishes, and evidence suggest that the energy density of adults would be even higher in fully grown individuals (Van de Putte et al. 2010). This suggestion is supported by a relatively high energetic density of larger fish from the Ross Sea (Lenky et al. 2012). This increased energy density could be a result of increased lipid content, which increases with age and size. This increase is suggested to be needed for buoyancy, to compensate for increasing weight, rather than an energy storage, as it is assumed that sufficient copepod and euphausiid prey are available for *Pleuragramma antarctica* year-round, and because large lipid stores were still found in this fish after winter (Gon and Heemstra 1990; Friedrich and Hagen 1994; Hubold and Hagen 1997). However, there is also evidence that *Pleuragramma antarctica* is cannibalistic from a study conducted in late spring (Eastman 1985). The difference in energy density between juvenile and adult fish can also be explained by the higher investment in protein growth rather that lipid accumulation, which is a common phenomenon in fish (Shul’man 1974). No data on energy density are available for the spawning season, presumably occurring in winter and spring, with a possible extended season into December in the Ross Sea (Vacchi et al. 2004).

#### Other nototheniidae

High energy densities of 29.9 and 29.4 kJ g^−1^ DW were reported for *Dissostichus mawsoni* (Antarctic toothfish) and *Dissostichus eleginoides* (Patagonian toothfish), respectively (Durand and Nicolle 1980; Lenky et al. 2012). *Dissostichus mawsoni* occurs mainly in high Antarctic waters. *Dissostichus eleginoides* is more distributed in the northern parts of the Southern Ocean, particularly around the sub-Antarctic islands, and around the southern tip of South America (Duhamel et al. 2014). A significant proportion of the diets of *Dissostichus* spp. consist of other fish (Kock 1992). *Dissostichus* spp. is of great commercial interest and is harvested using longlines. All notothenioids lack a swim bladder. Most species are heavier than sea water, but still relatively light in weight compared to other teleosts (Eastman and DeVries 1982). Together with *Pleuragramma antarctica* and likely *Aethotaxis mitopteryx*, *D. mawsoni* accumulates lipids to achieve neutral buoyancy (Eastman and DeVries 1982; Kock 1992; Lenky et al. 2012). Juvenile *D. mawsoni* gradually becomes more buoyant with increasing size until they reach neutral buoyancy with adulthood at an approximate length of 81 cm SL (Near et al. 2003).

The energy density of other nototheniid species found in the literature ranged from 18.6 kJ g^−1^ DW for *Trematomus scotti* to 26.8 kJ g^−1^ DW for *Trematomus lepidorhinus* (proximate composition, Lenky et al. 2012), both caught in the Ross Sea during summer. Of the species listed in Lenky et al. (2012), *Lepidonotothen squamifrons*, *Trematomus bernacchii*, *Trematomus hansoni*, *Trematomus pennelli* and *T. scotti* are benthic species (Eastman and DeVries 1982; Lenky et al. 2012). Therefore, they are suggested to have less lipids and a higher proportion of ash (Hagen et al. 2000; Lenky et al. 2012). Furthermore *Trematomus* spp., *Notothenia coriiceps* and *Gobionotothen gibberifrons* mainly feed on benthic organisms which can have a relatively low energetic value such as polychaetes, molluscs and amphipods (Kock 1992; Lenky et al. 2012). *Trematomus lepidorhinus* feeds away from the bottom and possibly has more fat to increase buoyancy, explaining its higher energetic density (Lenky et al. 2012), although *L. squamifrons* has also been suggested to feed on both benthic and pelagic organisms (Kock 1992). Similar to *Pleuragramma antarctica*, the lipid content of *T. lepidorhinus* is known to increase with increasing size and weight (Friedrich and Hagen 1994).

*Champsocephalus gunnari* and *Chaenocephalus aceratus* have a northerly distribution usually occurring close to the APF, while the distribution of *Channichthys* spp. is limited to the Kerguelen Plateau (Duhamel et al. 2014). These species have similar energetic densities while they utilize different food sources (Kock 1992). An overview of recorded average energy density measurements of nototheniid fish species including, where possible, values expressed in kJ g^−1^ WW can be found in Table 3. Due to recent changes in the classification, former separate families are now included in the family Nototheniidae and the new proposed sub-families of the fish are given in brackets in the table (Duhamel et al. 2014). The energy densities of gonad, liver and muscle tissue of several nototheniid fish were measured separately using bomb calorimetry by Vanella et al. (2005). In most investigated species, the AFDW energy densities were highest in the liver (Vanella et al. 2005).

**Table 3:** Overview of the average energy density of several nototheniid species

Season	Location	*n*	Mean size (mm)	Water content (% WW)	Mean energy density		Method	Source
					kJ g^−1^ WW	kJ g^−1^ DW		
*Champsocephalus gunnari* (Channichthyinae)								
Autumn	Kerguelen	3	311.7 ± 16.1 SD	76.7 ± 2.0 SD	5.4 ± 0.3 SD	23.2 ± 0.6 SD	BC	Lea et al. (2002)
	Scotia Sea	3	437^a^ ± 15 SD	81.0 ± 0.4 SE	*4.65*	*24.74*	PC^b,c^	Oehlenschläger (1991)
Spring/summer	Kerguelen			80.1	*4.74*	*23.84*	PC^c^	Durand and Nicolle (1980)
*Chaenocephalus aceratus* (Channichthyinae)								
Autumn	Scotia Sea	10	497^a^ ± 34 SD	81.2 ± 0.8 SE	*4.56*	*24.24*	PC^b,c^	Oehlenschläger (1991)
*Channichthys rhinoceratus* (Channichthyinae)								
Spring	Kerguelen			82.8	*3.97*	*23.09*	PC^c^	Durand and Nicolle (1980)
*Dissostichus mawsoni* (Dissostichinae)								
Spring	McMurdo	1		68.6	9.4	*29.94*	BC	Lenky et al. (2012)
*Dissostichus eleginoides* (Dissostichinae)								
Spring/summer	Kerguelen			69.4	*9.00*	*29.42*	PC^c^	Durand and Nicolle (1980)
*Pagothenia borchgrevinki* (Trematominae)								
Spring	McMurdo 2006	1 (p)	182^a^ ± 3 SE	77.2	5.6	*24.56*	BC	Lenky et al. (2012)
	McMurdo 2006	4	205^a^± 26 SE	77.6 ± 3.1 SE	5.3 ± 1.3 SE	*23.66*	BC	Lenky et al. (2012)
	McMurdo 2007	4	235^a^ ± 27 SE	76.0 ± 2.5 SE	5.7 ± 1.1 SE	*23.75*	BC	Lenky et al. (2012)
*Trematomus bernacchii* (Trematominae)								
Spring	McMurdo 2006	(p)	146^a^ ± 18 SE	78.3	4.7	*21.66*	BC	Lenky et al. (2012)
	McMurdo 2007	(p)	164^a^ ± 25 SE	77.4	5.0	*22.12*	BC	Lenky et al. (2012)
	McMurdo 2007	4	189^a^ ± 22 SE	76.2 ± 3.0 SE	5.5 ± 1.3 SE	*23.11*	BC	Lenky et al. (2012)
*Trematomus hansoni* (Trematominae)								
Spring	McMurdo Sound	7	211^a^ ± 262 SE	76.7 ± 2.0 SE	5.4 ± 0.9 SE	*23.18*	BC	Lenky et al. (2012)
*Trematomus pennellii* (Trematominae)								
Spring	McMurdo Sound	1 (p)	141^a^ ± 16 SE	78.3	4.6	*21.20*	BC	Lenky et al. (2012)
*Trematomus eulepidotus* (Trematominae)								
Summer	Ross Sea	(p)	196^a^ ± 31 SE	75.6	5.7	*23.36*	BC	Lenky et al. (2012)
*Trematomus lepidorhinus* (Trematominae)								
Summer	Ross Sea	(p)	274^a^ ± 56 SE	71.3	7.7	*26.83*	BC	Lenky et al. (2012)
*Trematomus scotti* (Trematominae)								
Summer	Ross Sea	(p)	129^a^ ± 7 SE	78.5	4.0	*18.60*	BC	Lenky et al. (2012)
*Lepidonotothen squamifrons* (Trematominae)								
Summer	Ross Sea	(p)	224^a^ ± 317 SE	81.3	4.0	*21.39*	BC	Lenky et al. (2012)
Spring/summer	Kerguelen			79.8	*4.78*	*23.67*	PC^c^	Durand and Nicolle (1980)
					5.00			Goldsworthy et al. (2001)
*Notothenia rossi* (Nototheniinae)								
Spring	Kerguelen			76.7	*6.07*	*26.07*	PC^c^	Durand and Nicolle (1980)
*Notothenia neglecta* (Nototheniinae)								
Autumn	Scotia Sea	3	317^a^ ± 51 SD	78.4 ± 1.0 SE	*5.35*	*24.77*	PC^b,c^	Oehlenschläger (1991)
*Gobionotothen gibberifrons* (Gobinototheninae)								
Autumn	Scotia Sea	13	377^a^ ± 17 SD	79.8 ± 0.4 SE	*4.85*	*24.05*	PC^b,c^	Oehlenschläger (1991)

Sub-families are given in brackets. Energy densities were measured using bomb calorimetry (BC) and proximate composition (PC). Energy densities in italics represent values that were converted using information from the given sources. *n* represents the number of samples measured. Where this expresses samples of pooled individuals, this is indicated with (p). The standard error (SE) or standard deviation (SD) as given in the original source is added where available (±). The mean size is given in standard length (SL) unless otherwise indicated

^a^Measured in total length (TL)

^b^Crude protein measurement used

^3^Carbohydrates not measured

#### *Electrona antarctica*

*Electrona antarctica* is a circumpolar, widely distributed mesopelagic species found at and south of the APF (Duhamel et al. 2014). It has been found to be an important prey species for flying birds in the Weddell and Scotia Seas (Ainley et al. 1991). Records of the average energy density of *E. antarctica* showed a range between 18.9 kJ g^−1^ DW, for fish from the Scotia Sea during spring (proximate composition, Donnelly et al. 1990), and 34.3 kJ g^−1^ DW, for fish from the Kerguelen plateau during winter (bomb calorimetry; Lea et al. 2002). The lower range of values found in the literature were usually estimates made using proximate composition. Average recorded energy density measurements of *E. antarctica* including, where possible, values expressed in kJ g^−1^ WW are listed in Table 4.

**Table 4:** Overview of the average energy densities of *Electrona antarctica*

Season	Location	*n*	Mean size (mm; SL)	WW (g)	DW (g)	Water content (%)	Mean energy density		Method	Source
							kJ g^−1^ WW	kJ g^−1^ DW		
Summer	Macquarie	20	50.4 ± 13.1 SD	1.9 ± 1.5 SD	0.6 ± 0.5 SD	69.9 ± 4.3 SD	9.04 ± 1.89 SD	30.76 ± 8.30 SD	BC	Tierney et al. (2002)
	Lazarev Sea	31	49.1 ± 16.8 SD^a^	1.4 ± 1.4 SD	0.5 ± 0.5 SD	73.3 ± 7.2 SD	9.94 ± 1.11 SD	32.26 ± 1.15 SD	BC	This study (PS89)
	WAP		76.5 ± 3.79 SE					31.9 ± 0.29 SE	BC	Ruck et al. (2014)
	Elephant Island	3				71.7 ± 0.6 SE	8.55 ± 0.19 SE^b^	*30.21*	PC	Ishii et al. (2007)
Autumn	East Antarctica	22	57.4 ± 21.2 SD	2.6 ± 2.5 SD	0.7 ± 0.8 SD	73.7 ± 4.0 SD	7.26 ± 1.68 SD	27.21 ± 2.76 SD	BC	Van de Putte et al. (2010)
	Lazarev Sea	113	47.6 ± 15.9 SD	1.8 ± 1.8 SD	0.6 ± 0.6 SD	68.4 ± 4.14 SD	9.35 ± 1.58 SD	29.4 ± 1.80 SD	BC	Van de Putte et al. (2006)
	Weddell Sea	27	61.9	3.9	*1.2*	68.7 ± 3.4 SD	6.73	*21.5*	PC	Donnelly et al. (1990)^c^
						68.2	9.11	*28.65*	BC	Green and Gales (1990)
Winter	Lazarev Sea	74	52.6 ± 19.5 SD	2.4 ± 3.5 SD	0.8 ± 1.2 SD	71.3 ± 4.2 SD	8.35 ± 1.82 SD	28.77 ± 2.67 SD	BC	Van de Putte (2008)
	Kerguelen	5	64.5 ± 8.6 SD	3.2 ± 1.8 SD	*1.3*	60.8 ± 8.8 SD	13.3 ± 2.6 SD	34.3 ± 3.8 SD	BC	Lea et al. (2002)
	Scotia Sea	35	68.3	5.6	*1.7*	69.6 ± 3.7 SD	7.71	*25.36*	PC	Donnelly et al. (1990)
Spring	Ross Sea	(p)	81^a^ ± 10 SE	7.4 ± 2.5 SE	*2.3*	69.6	9.0	*29.61*	BC	Lenky et al. (2012)
	Scotia Sea	16	66.1	3.8	*1.2*	69 ± 3.7 SE	5.86	*18.9*	PC	Donnelly et al. (1990)

In the ‘method’ column the method used for energetic value determination is indicated, where BC is bomb calorimetry and PC is proximate composition. Numbers in italics represent values that were converted using the energetic values, wet weights, dry weights and water contents from the given sources. *n* represents the number of samples measured. Where this expresses samples of pooled individuals, this is indicated with (p). The standard error (SE) or standard deviation (SD) as given in the original source is added where available. The mean size is given in standard length (SL) unless otherwise indicated

^a^Measured in total length (TL)

^b^A lipid factor of 39.6 kJ g^−1^ used for energy density estimation

^c^A factor of 4.19 was used to convert calories to joules

**Fig. 2:**
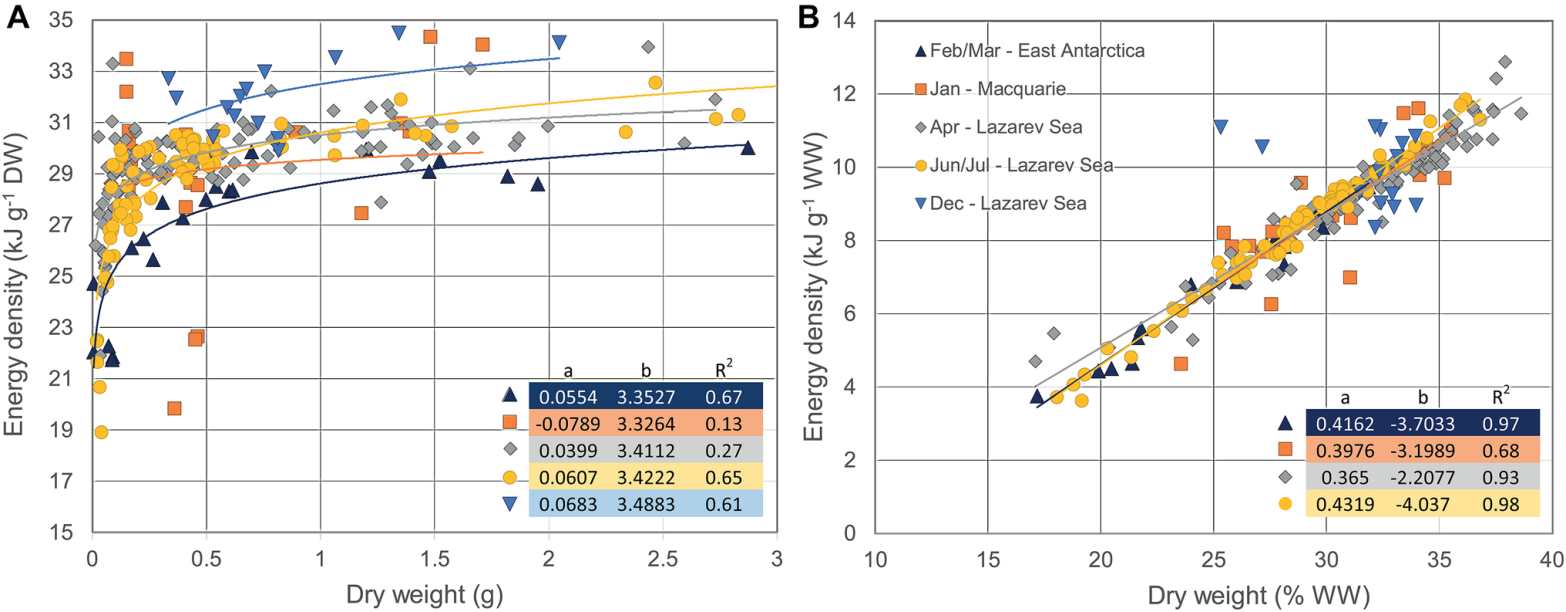
*Electrona antarctica*; **a** the relationship between dry weight (DW) and energy density g^−1^ DW including the parameters for the linear regression of ln(*y*) = *a* + *b*ln(*x*), and the corresponding power function *y* = *x*^*b*^ *e*^*a*^ and, **b** the relationship between DW (%WW) and energy density g^−1^ wet weight (WW) including regression parameters of the linear regression lines *y* = *ax* + *b*. Regression parameters are depicted in the figures. Data were obtained from Tierney et al. (2002) (Macquarie Island), Van de Putte et al. (2010) (East Antarctica, February–March), Van de Putte et al. (2006) (Lazarev Sea, April), Van de Putte 2008 (Lazarev Sea, June/July) or collected during PS89 (Lazarev Sea, December). All measurements were done using bomb calorimetry. The legend, depicted in B, indicates month and location of data collection. No regression was fitted for the December-Lazarev Sea data in **b**, due to two individuals that had divergent dry weights

The energy content of *E. antarctica* generally increased with increasing size (Donnelly et al. 1990; Van de Putte et al. 2006, 2010). Van de Putte et al. (2006) showed that the energy density of *E. antarctica* strongly increased with size in age class 0, and slows down from the second year onward while the variation increases. This trend is confirmed in fish from East Antarctica and the Lazarev Sea in several seasons (Fig. 2a). This size–energy density relationship suggests that the small fish invest more of their energy in growth compared to the older individuals, probably due to the need to grow quickly to avoid predation (Van de Putte et al. 2006).

Donnelly et al. (1990) found an increase in lipid and energy content from spring to autumn, and from autumn to winter (Table 4), and suggested that this might be due to the accumulation of reserves for winter and early spring. In contrast, however, the data from the Lazarev Sea suggest highest energy densities in summer, decreasing towards autumn and winter. In general, energy density of *E. antarctica* was higher in the Lazarev Sea compared to East Antarctica and Macquarie Island (Fig. 2a). Available measurements of individual fish, depicted in Fig. 2, allowed for a statistical comparison. The energy density of fish from the Lazarev Sea in summer was significantly higher than all other data (ANOVA *F*(24, 254) = 36.8, *p* < 0.001; Tukey’s HSD, *p* < 0.0001), while the energy density of fish caught in East Antarctica in autumn was significantly lower than all other locations (Tukey’s HSD, *p* < 0.03). Based on current available science, *E. antarctica* is assumed to spawn year-round with a peak in late summer/early autumn, or late spring/summer (Donnelly et al. 1990). In contrast, Gon and Heemstra (1990) suggested a peak spawning season in autumn/winter. However, the energetic content of maturing gonads does not appear to contribute significantly to the total energy content of the fish (Donnelly et al. 1990). Therefore, the main driver for differences in energy density is probably food composition, which differs for *E. antarctica* depending on area and season (Flores et al. 2008). The relationship between DW (in %WW) and wet weight energy density was similar in fish from all seasons and regions (ANCOVA, *p* > 0.05; Fig. 2b).

#### *Gymnoscopelus braueri*

*Gymnoscopelus braueri* is also a circumpolar, widely distributed species found between de SAF and the SACCF (Duhamel et al. 2014). Recorded average energy densities of *G. braueri* ranged from 19.9 kJ g^−1^ DW in fish from the Scotia Sea during spring (proximate composition, Donnelly et al. 1990) to 39.0 kJ g^−1^ DW in fish from the vicinity of Macquarie Island during summer (bomb calorimetry, Tierney et al. 2002). An overview of recorded average energy density measurements of *G. braueri* including, where possible, values expressed in kJ g^−1^ WW can be found in Table 5.

**Table 5:** Overview of the average energetic densities of *Gymnoscopelus braueri*

Season	Location	*n*	Mean size (mm)	WW (g)	DW (g)	Water content (%)	Mean energy density		Method	Source
							kJ g^−1^ WW	kJ g^−1^ DW		
Summer	South Georgia	3				66.1 ± 1.5 SE	9.06	*29.85*	PC	Clarke and Prince (1980)
	Macquarie	18	78.2 ± 35.3 SD	5.3 ± 5.7 SD	1.9 ± 2.2 SD	69.4 ± 8.4 SD	10.91 ± 1.51 SD	39.03 ± 14.33 SD	BC	Tierney et al. (2002)
Autumn	Weddell Sea	3	101.3	8.7	*2.9*	66.6 ± 2.2 SD	7.94	*23.77*	PC	Donnelly et al. (1990)^b^
	Lazarev Sea	20	87.3 ± 18.1 SD	6.3 ± 5.3 SD	1.9 ± 1.7 SD	69.5 ± 4.0 SD	8.86 ± 1.42 SD	29.37 ± 1.51 SD	BC	Van de Putte et al. (2006)
Winter	Scotia Sea	23	81.2	5.8	*1.9*	67.2 ± 2.3 SE	7.52	*22.93*	PC	Donnelly et al. (1990)
Winter/spring	Weddell Sea	3 (p)	49.7 ± 9.0 SD^1^	0.7 ± 0.5 SD	0.3 ± 0.2 SD	62.1 ± 2.0 SD	10.68 ± 0.24 SD	29.14 ± 1.31 SD	BC	This study (PS81)
Spring	Ross Sea	(p)	101^1^ ± 7 SE	9 ± 1.9 SE	*2.8*	68.5	9.3	*29.52*	BC	Lenky et al. (2012)
	Scotia Sea	3	110.3	9.2	*3.3*	64.2 ± 2.5 SD	7.14	*19.94*	PC	Donnelly et al. (1990)

Energy densities are measured using bomb calorimetry (BC) and proximate composition (PC). Energetic density in italics represent values that were converted information from the given sources. *n* represents the number of samples measured. Where this expresses samples of pooled individuals, this is indicated with (p). The standard error (SE) or standard deviation (SD) as given in the original source is added where available. The mean size is given in standard length (SL) unless otherwise indicated

^a^Measured in total length (TL)

^b^A factor of 4.19 was used to convert calories to joules

**Fig. 3:**
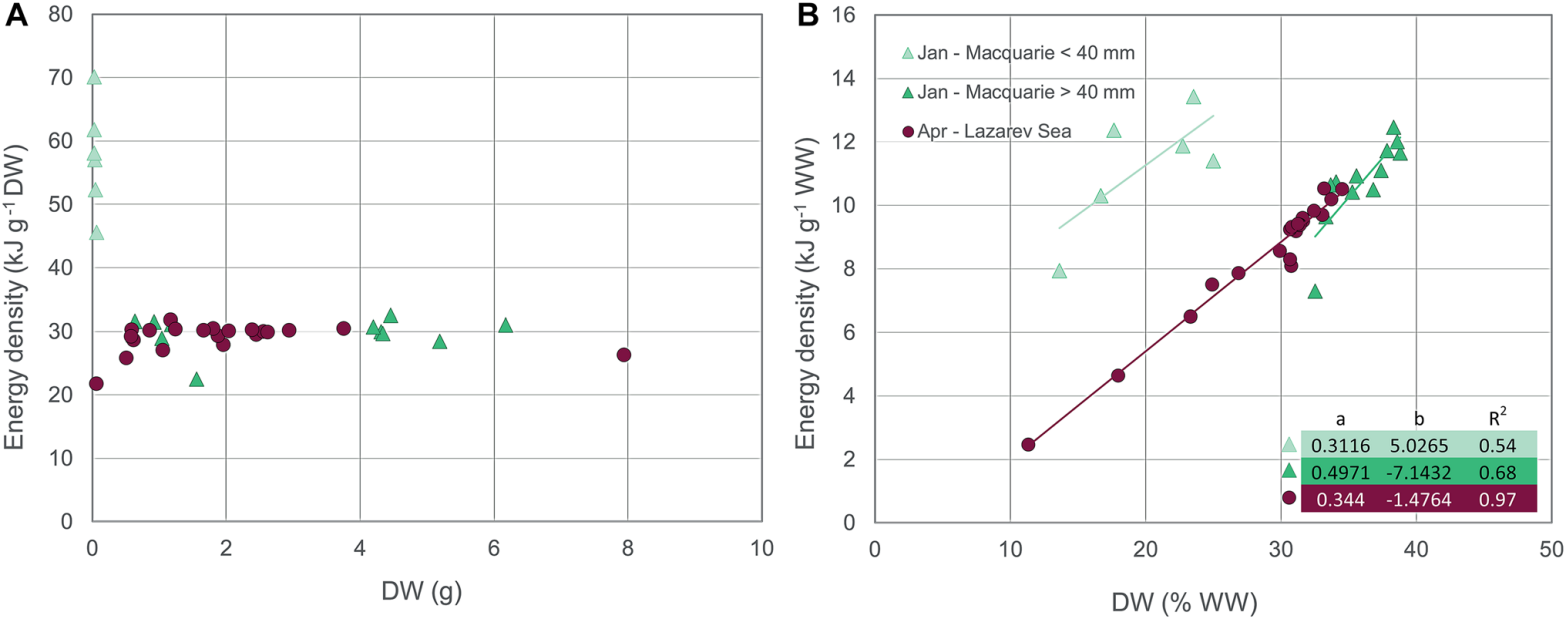
*Gymnoscopelus braueri*; **a** the relationship between dry weight (DW) and energy density g^−1^ DW and, **b** the relationship between DW (%WW) and energy density g^−1^ wet weight (WW) including parameters of the linear regression lines *y* = *ax* + *b*. Regression parameters are depicted in the figure. Data were obtained from Tierney et al. (2002) (Macquarie Island, January) and Van de Putte et al. (2006) (Lazarev Sea, April). Due to significant differences in energetic density, data from Tierney et al. (2002) were separated in individuals < 40 and > 40 mm. All measurements were done using bomb calorimetry. Legend indicates month and location of data collection

Tierney et al. (2002) found a strong difference in calorific value between size classes in summer. Fish < 40 mm had a significantly higher dry weight energy density compared to larger individuals, which is in contrast to *E. antarctica*. Interestingly, the small fish also had a significantly higher water content (Tierney et al. 2002). This pattern was, however, not confirmed by data from the Lazarev Sea in autumn where the dry-weight energy density did not differ in different sized fish (Van de Putte et al. 2006). Within the size classes found in Tierney et al. (2002), there was no (linear) relationship between size and dry-weight energy density (Fig. 3a). The data from Macquarie Island (Tierney et al. 2002) and the Lazarev Sea (Van de Putte et al. 2006 and PS81) allowed for statistical comparison, which showed that the energy density of *G. braueri* > 40 mm did not vary significantly between seasons and regions, even in the relatively small fish from winter (ANOVA, *F*(3,35) = 0.288, *p* = 0.83).

In fish > 40 mm, the relationship between water content and wet-weight energy density of *G. braueri* from the Lazerev Sea in April and the Macquarie region in January show similar slopes (Fig. 3b) suggesting that there is no evidence that tissues replacing the body water are markedly different between seasons and/or regions (ANCOVA, *p* > 0.05). As the small fish from the Macquarie region have a relatively high energy density, the intercept of this regression is significantly higher compared to regressions of the other data (ANCOVA, *p* < 0.05).

#### Other myctophids

The average energy density of other myctophid species reported in the literature range from 17.1 kJ g^−1^ DW of *Protomyctophum tenisoni* and *Protomyctophum bolini* caught in the Scotia Sea during winter (proximate composition, Donnelly et al. 1990) to 39.3 kJ g^−1^ DW of *Protomyctophum andriashevi* caught in the vicinity of Macquarie Island during summer (bomb calorimetry, Tierney et al. 2002). Similar to *G. braueri*, Tierney et al. (2002) found several, but not all, other myctophid species in which small individuals (< 40 mm SL, approximately) had a significantly higher dry-weight energy density such as *Gymnoscopelus fraseri*, *P. andriashevi*, *P. bolini* and *Lampanyctus archirus*. In contrast to the other species, the water content of *G. fraseri* and *P andriashevi* did not differ significantly between size classes (Tierney et al. 2002). An overview of recorded average energy density measurements of myctophid fish species including, where possible, values expressed in kJ g^−1^ WW is listed in Table 6.

**Table 6:** Overview of the average energy density of several myctophid species

Season	Location	*n*	Size (mm)	Water content (% WW)	Mean energetic density		Method	Source
					kJ g^−1^ WW	kJ g^−1^ DW		
*Gymnoscopelus ophistopterus*								
Autumn	Weddell Sea	6	108.8	80.1 ± 3.3 SD	4.58	*23.02*	PC	Donnelly et al. 1990^d^
*Gymnoscopelus fraseri*								
Summer	Macquarie	18 (p)	35–78	73.1 ± 4.0 SD	*7.89*	29.32 ± 8.62 SD	BC	Tierney et al. (2002)
Winter	Kerguelen	5	66.2 ± 7.1 SD	62.6 ± 10.1 SD	10.2 ± 3.5 SD	27.0 ± 2.9 SD	BC	Lea et al. (2002)
*Gymnoscopelus piabilis*								
Winter	Kerguelen	5	187.6 ± 32.0 SD	68.5 ± 3.0 SD	9.5 ± 1.7 SD	30.0 ± 30.0 SD	BC	Lea et al. (2002)
*Gymnoscopelus nicholsi*								
Summer	Elephant Island	3		76.7 ± 0.7 SE	5.82 ± 0.22 SE^a^	*24.98*	PC	Ishii et al. (2007)
Autumn				67	8.43	*25.55*	PC	VNIRO (2000)
				66.4	9.58	*28.51*	BC	Green and Gales (1990
Winter	Kerguelen	1	128	66.8	9.80	28.00	BC	Lea et al. (2002)
	Scotia Sea	1	148	59.6	11.75	*29.08*	PC	Donnelly et al. (1990)
Spring	Ross Sea	(p)	149^b^ ± 7 SE	64.9	10.3	*29.34*	BC	Lenky et al. (2012)
*Gymnoscopelus microlampas*								
Summer	Macquarie	6 (p)	84–122	74.7 ± 1.3 SD	*5.72*	22.62 ± 1.14 SD	BC	Tierney et al. (2002)
*Electrona subaspera*								
Summer	Macquarie	6 (p)	10–117	72.1 ± 1.7 SD	*7.41*	26.56 ± 1.15 SD	BC	Tierney et al. (2002)
Winter	Kerguelen	3	92.7 ± 7.5 SD	72.3 ± 1.6 SD	7.4 ± 1.0 SD	26.6 ± 2.1 SD	BC	Lea et al. 2002
*Electrona carlsbergi*								
Summer	South Georgia	3		71.2 ± 0.3 SE	6.57	*22.84*	PC	Clarke and Prince (1980)
				72.7	5.87	*21.50*	PC	VNIRO (2000)
	Possession Island	3	78.8 ± 4.6 SD	70.2 ± 0.4 SD	7.0 ± 0.2 SD^c^	23.5 ± 0.4 SD^c^	BC	Cherel and Ridoux (1992)
	Elephant Island	3		73.8 ± 0.7 SE	6.92 ± 0.13 SE	*26.41*	PC	Ishii et al. (2007)
	Macquarie	6 (p)	26–97	76.7 ± 5.2 SD	*5.05*	21.67 ± 3.17 SD	BC	Tierney et al. (2002)
Winter	Kerguelen	6	84.7 ± 3.6 SD	67.0 ± 3.2 SD	8.6 ± 1.2 SD	25.9 ± 3.2 SD	BC	Lea et al. (2002)
Spring	Ross Sea	(p)	72 ± 6 SE^b^	73.9	6.1	*23.37*	BC	Lenky et al. (2012)
*Krefftichthys anderssoni*								
Summer	Possession Island	2	47.7 ± 9.2 SD	69.3 ± 1.4 SD	8.1 ± 0.3 SD^c^	26.4 ± 0.1 SD^c^	BC	Cherel and Ridoux (1992)
	Macquarie	18 (p)	40–69	69.8 ± 1.9 SD	*8.32*	27.54 ± 2.75 SD	BC	Tierney et al. (2002)
Autumn				66.6	10.12	30.30	BC	Green and Gales (1990)
*Protomyctophum tenisoni*								
Summer	Macquarie	6 (p)	43–51	73.2 ± 1.1 SD	*5.50*	20.53 ± 0.65 SD	BC	Tierney et al. (2002)
Winter	Kerguelen	1	45	74.6	6.1	24.2	BC	Lea et al. (2002)
	Scotia Sea	3	47	72.2 ± 0.6 SD	4.75	*17.09*	PC	Donnelly et al. 1990
*Protomyctophum andriashevi*								
Summer	Macquarie	12 (p)	23–51	75.7 ± 5.3 SD	*9.54*	39.26 ± 21.48 SD	BC	Tierney et al. (2002)
*Protomyctophum bolini*								
Summer	Macquarie	18 (p)	29–61	73.5 ± 3.9 SD	*7.42*	28.00 ± 10.61 SD	BC	Tierney et al. (2002)
Winter	Scotia Sea	6	48.3	74.6 ± 1.4 SD	4.34	*17.09*	PC	Donnelly et al. (1990)
*Protomyctophum parallelum*								
Summer	Macquarie	6 (p)	20–48	70.9 ± 3.6 SD	*8.23*	28.27 ± 12.28 SD	BC	Tierney et al. (2002)
*Lampanyctus archirus*								
Summer	Macquarie	18 (p)	35-147	78.5 ± 3.4 SD	*6.12*	28.47 ± 14.43 SD	BC	Tierney et al. (2002)

Energy density measurement were done using bomb calorimetry (BC) and proximate composition (PC). Energy densities in italics represent values that were converted using information from the given sources. *n* represents the number of samples measured. Where this expresses samples of pooled individuals, this is indicated with (p). The standard error (SE) or standard deviation (SD) as given in the original source is added where available. The mean size is given in standard length (SL) unless otherwise indicated

^a^A lipid factor of 39.6 kJ g^−1^ used for energy density estimation

^b^Measured in total length (TL)

^c^Sample taken from bird stomach contents, in which the energetic value is potentially overestimated due to water removal in stomach

^d^A factor of 4.19 was used to convert calories to joules

Of the species listed, *P. tenisoni*, *Electrona carlsbergi*, *G. fraseri* and *Gymnoscopelus piabilis* occur mainly in the sub-Antarctic zone, while the other species occur south of the PF or have a more wide distribution. *Protomyctophum tenisoni*, *E. carlsbergi*, *Gymnoscopelus ophistopterus*, and *Gymnoscopelus microlampas* have relatively low energy densities considering what can be assumed for lipid-rich myctophid species. Lea et al. (2002) found that *P. tenisoni* had a relatively low lipid content compared to other investigated myctophid fishes. *Electrona carlsbergi* was, however, lipid-rich in this study (Lea et al. 2002).

#### *Bathylagus antarcticus*

Of the two main species of Bathylagidae (*Bathylagus tenuis* and *Bathylagus antarcticus*) found in the meso- and bathypelagic zones of the Southern Ocean, *B. antarcticus* has the more southern distribution (Duhamel et al. 2014). Recorded average energy densities of *B. antarcticus* ranged from 14.8 kJ g^−1^ DW, estimated in fish from the winter Scotia sea using proximate composition (Donnelly et al. 1990), to 22.8 kJ g^−1^ DW measured in fish from the spring Ross Sea using bomb calorimetry (Lenky et al. 2012). Average recorded energy density measurements of *B. antarcticus* including, where possible, values expressed in kJ g^−1^ WW are listed in Table 7.

**Table 7:** Overview of the average energetic densities of *Bathylagus antarcticus*

Season	Location	*n*	Mean size (mm)	WW (g)	DW (g)	Water content (%)	Mean energy density		Method	Source
							kJ g^−1^ WW	kJ g^−1^ DW		
Summer	Macquarie	18	116.8 ± 35.4 SD	14.2 ± 14.2 SD	2.7 ± 2.8 SD	81.7 ± 1.9 SD	3.93 ± 1.17 SD	21.43 ± 4.88 SD	BC	Tierney et al. (2002)
Autumn	Lazarev Sea	7	77.6 ± 23.4 SD	3.1 ± 3.6 SD	0.4 ± 0.4	85.6 ± 2.5 SD	2.92 ± 0.42 SD	20.36 ± 1.32 SD	BC	Van de Putte et al. (2006)
	Weddell Sea	32	77.2	3.8	*0.5*	85.9 ± 2.0 SD	2.24	*15.89*	PC	Donnelly et al. (1990)^a^
Winter	Scotia Sea	16	90	7.8	*0.9*	88.4 ± 1.4 SD	1.72	*14.83*	PC	Donnelly et al. (1990)
Spring	Ross Sea	(p)	151^a^ ± 20 SE	38.6 ± 18.2 SE	*4.9*	87.3	2.9	*22.83*	BC	Lenky et al. (2012)
	Scotia Sea	8	99.4	5.8	*0.9*	85.1 ± 2.1 SD	2.22	*14.89*	PC	Donnelly et al. (1990)

Energy density measurements were done using bomb calorimetry (BC) and proximate composition (PC). Energetic values in italics represent values that were converted using information from the given sources. *n* represents the number of samples measured. Where this expresses samples of pooled individuals, this is indicated with (p). The standard error (SE) or standard deviation (SD) as given in the original source is added where available. The mean size is given in standard length (SL) unless otherwise indicated

^a^Measured in total length (TL)

^b^A factor of 4.19 was used to convert calories to joules

**Fig. 4:**
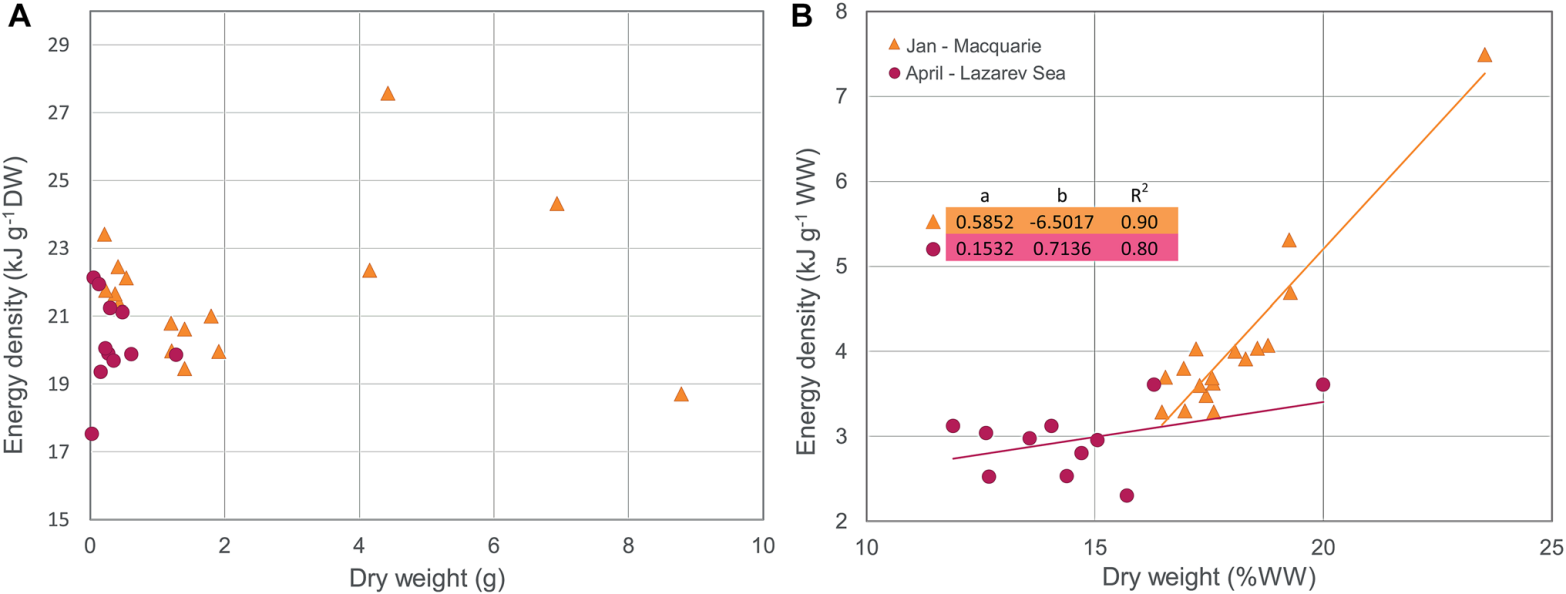
*Bathylagus antarcticus*; **a** the relationship between dry weight (DW) and dry-weight energy density and, **b** the relationship between DW (%WW) and energy density g^−1^ wet weight (WW) including parameters of the linear regression lines *y* = *ax* + *b*. Regression parameters are depicted in the figure. Data were obtained from Tierney et al. (2002) (Macquarie Island, January) and Van de Putte et al. (2006) (Lazarev Sea, April). All measurements were done using bomb calorimetry. Legend indicates month and location of data collection

The dry-weight energy density of *B. antarcticus* caught in the Lazarev Sea in April (Van de Putte et al. 2006) did not differ significantly from fish caught in the vicinity of Macquarie Island in January (Tierney et al. 2002), even though the latter fish were larger (Fig. 4a). In both seasons/regions, the energy density did not change with changing sizes. Water content of *B. antarcticus* was significantly higher in April than it was in January, resulting in a lower wet weight energy density in the Lazarev Sea in April compared to the Macquarie region in January. The relationship between wet-weight energy density and proportional dry-weight found by Van de Putte et al. (2006) suggested that water is replaced with low energy tissue. This relationship is, however, different in the fish from Tierney et al. (2002), where energy density is relatively low compared to other fish species from the same study, but the wet-weight energy density increases relatively fast with decreasing water content (Fig. 4b).

#### Other fishes

An overview of recorded average energy density measurements of five fish species other than the ones listed above including, where possible, values expressed in kJ g^−1^ WW can be found in Table 8. The families to which the species belong are given in the table. Among these fishes, *Paradiplospinus gracilis* had the highest mean energy density of 25.6 kJ g^−1^ DW. The lowest values were found in *Notolepis coatsi* from autumn and winter in the Weddell–Scotia Seas sector (14.9 and 15.6 kJ g^−1^ DW, respectively). Both measurements were done using proximate composition. Ciancio et al. (2007) list another nine species of which the distribution in the Southern Ocean is limited to the Patagonian shelf. Their energy density (measured using bomb calorimetry) ranged from 16.2 kJ g^−1^ DW for *Genypterus blacodes* (Ophidiidae) to 26.2 kJ g^−1^ DW for *Eleginops maclovinus* (Eleginopidae; Ciancio et al. 2007).

**Table 8:** Overview of the average energy density of several fish species

Season	Location	*n*	Mean size (mm)	Water content (% WW)	Mean energy density		Method	Source
					kJ g^−1^ WW	kJ g^−1^ DW		
*Notolepis coatsi (Paralepididae)*								
Autumn	Weddell Sea	5	62.4	82.2 ± 2.7	2.65	*14.89*	PC	Donnelly et al. (1990)^b^
Winter	Scotia Sea	5	63.4	79.4 ± 3.4	3.22	*15.63*	PC	Donnelly et al. (1990)
Summer	East Antarctica	3	168 ± 52	79.8 ± 1.3	4.42 ± 0.33	21.90 ± 0.73	BC	Van de Putte et al. (2010)
*Paradiplospinus gracilis* (Gempylidae)								
Summer	Possession Island	1	168.7	78.9	4.6^a^	21.8^a^	BC	Cherel and Ridoux **(**1992)
Winter	Scotia Sea	2	325.5	69.1 ± 2.4	7.92	*25.63*	PC	Donnelly et al. (1990)
*Antimora rostrata* (Moridae)								
Summer	Macquarie	2 (p)	227–225	80.1 ± 1.0	*4.33*	21.75 ± 2.28	BC	Tierney et al. (2002)
*Stomias gracilis* (Stomiidae)								
Summer	Macquarie	18 (p)	130–278	77.8 ± 3.1	*5.15*	23.20 ± 2.99	BC	Tierney et al. (2002)
*Micromesistius australis* (Gadidae)								
	Patagonia	3	140–150	78.5	4.54	*21.12*	BC	Ciancio et al. (2007)

Families are given in brackets. Energy density measurements were done using bomb calorimetry (BC) and proximate composition (PC). Energy densities in italics represent values that were converted using information from the given sources. *n* represents the number of samples measured. Where this expresses samples of pooled individuals, this is indicated with (p). The standard deviation (SD) is given where available. The mean size is given in standard length (SL)

^a^Sample taken from bird stomach contents, in which the energetic value is potentially overestimated due to water removal in stomach

^b^A factor of 4.19 was used to convert calories to joules

### Other species

#### Squid

Squid are often a part of, or even dominate in some seasons, the diet of many top predators (Klages 1989; Ainley et al. 1991; Cherel et al. 1996; Kirkman et al. 2000; Van Franeker et al. 2001). Therefore, an indication of their energy density is highly relevant in trophic and ecosystem studies. Although measurements of squid are limited, reported values suggest that the energy density of squid increases with increasing latitudes (from the tropics to Southern Ocean), and that the energy density of squid in the Southern Ocean is comparable with that of nototheniid fish. Squid are difficult to catch with scientific sampling gear (Rodhouse et al. 2014), explaining the limited amount of measurements on this group (Table 9). Therefore, we have included some energetic density measurements from regions other than the Southern Ocean in this section for comparison.

**Table 9:** Overview of the average energy density of Southern Ocean squid species

Season	Location	*n*	Body length (mm)	Water content (%)	Mean energy density		Method	Source
					kJ g^−1^ WW	kJ g^−1^ DW		
*Doryteuthis gahi*								
				80.9	3.09^a^	*16.18*	PC	Pandit and Magar (1972)
	Patagonia	8	60–90	76.6	4.95	*21.16*	BC	Ciancio et al. (2007)
*Moroteuthis ingens*								
Summer	Possession Island	1		76.0	5.6^b^	23.51^b^	BC	Cherel and Ridoux (1992)
	New Zealand	6	356	80.3	*4.73* ^c^	*24.02* ^c^	PC	Vlieg (1984)
*Illex argentinus*								
	Patagonia	4	210–415	76.7	5.01	*21.52*	BC	Ciancio et al. (2007)

In the ‘method’ column the method used for energetic value determination is indicated, where BC is bomb calorimetry and PC is proximate composition. Energetic values in italics represent values that were converted using the energetic values, wet weights and dry weights from the given source. *n* represents the number of samples measured

^a^Based on measurements of water content, lipids (× 39.7 kJ g^−1^) and crude protein

^b^Mantle and tentacles

^c^Based on crude protein

Croxall and Prince (1982) provide an overview of energy densities of cephalopods from different locations. The reported values ranged from 14.9 to 19.9 kJ g^−1^ DW. The cephalopods listed in Croxall and Prince (1982) belong to the families Loliginidae, Octopodidae, Ommastrephidae, Onychoteuthidae and Sepiidae. Of the reported species, only the squid *Doryteuthis gahi* occurs south of the STF, over the Patagonian shelf in the sub-Antarctic region (Rodhouse et al. 2014). It had an energy density of 16.2 kJ g^−1^ DW (Ferreyra in Pandit and Magar 1972). Ciancio et al. (2007) reported an energy density of 21.2 kJ g^−1^ DW for *D. gahi*. They, furthermore, reported the energy density of *Illex argentinus*, also caught over the Patagonian shelf (Table 9; Ciancio et al. 2007).

*Moroteuthis ingens* is a very abundant species in the Southern Ocean. The mantle and tentacles of *M. ingens*, collected from the stomach contents of king penguins (*Aptenodytes patagonicus*) at Possession Island in summer, had an energy density of 23.5 kJ g^−1^ DW, measured using bomb calorimetry (Cherel and Ridoux 1992). Proximate composition values of *M. ingens* caught near New Zealand (Vlieg 1984) result in an estimated energy density of 24.0 kJ g^−1^ DW. The mantle, fins and tentacles of *M. ingens*, had a similar energy density of approximately 23 kJ g^−1^ DW. The energy density of the inner organs was higher (25.7 kJ g^−1^ DW), which is probably caused by ingested food residing in the stomach (Vlieg 1984) or lipids stored in the digestive gland (Phillips et al. 2001).

Two species of squid that are not known to reside in sub-Antarctic or Antarctic waters (Rodhouse et al. 2014), but which have been found in the stomachs of penguin species, had energy densities of 24.7 kJ g^−1^ DW (*Sepiotheuthis australis*) and 23.4 kJ g^−1^ DW (*Nototodarus gouldi*; Green and Gales 1990).

Clarke et al. (1985) measured the energy density of several species of squid caught in the north–east Atlantic Ocean. The energy value ranged from 17.5 kJ g^−1^ DW (1.8 kJ g^−1^ WW; *Mastigoteuthis* sp.) to 21.5 kJ g^−1^ DW (2.7 kJ g^−1^ WW; *Histioteuthis* sp.). The energy value per gram WW was highly variable due to different types of buoyancy regulation used by different squid species, resulting in large differences in water content between species. This did, however, not result in large differences in the energy density per gram DW, the range of which was limited (Clarke et al. 1985).

#### Gelatinous zooplankton

**Fig. 5:**
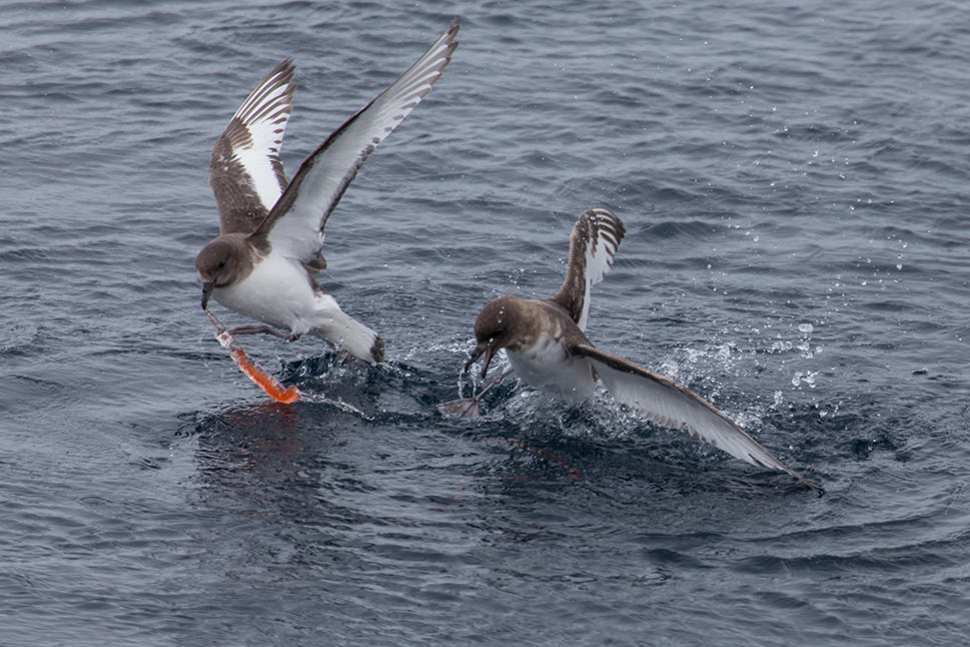
Antarctic Petrels (*Thalassoica antarctica*) feeding on gelatinous species in the Lazarev Sea during summer (© Jan Andries van Franeker)

A large biomass component of marine ecosystems is formed by gelatinous zooplankton (McInnes et al. 2017). The gelatinous zooplankton includes for instance Ctenophora, or comb jellies, and Cnidaria, including Scyphozoa and Hydrozoa. The latter class contains the order Siphonophora from which species such as *Diphyes antarctica* can dominate the epipelagic layer particularly during autumn and winter (Flores et al. 2014). Gelatinous species have often been viewed as an unimportant prey item for many organisms, due to both their low energetic value and the difficulty in detecting gelatinous prey with conventional diet assessments methods (e.g. stomach content analysis, leading to potential underestimation of their prevalence as a prey item; McInnes et al. 2017). However, they have been found to be more than an incidental part of the diet of many larger animals (Fig. 5), including albatrosses and Adélie penguins (*Pygoscelis adeliae*) in the Southern Ocean (Jarman et al. 2013; Thiebot et al. 2016; McInnes et al. 2017; Thiebot et al. 2017;). Although secondary ingestion cannot be excluded when using DNA analysis, results suggest that they are common prey item (Jarman et al. 2013; McInnes et al. 2017). Video observations captured Adélie penguins feeding on jellyfish, even when other preys were available (Thiebot et al. 2016, 2017). Certain jellyfish species are regularly invested with parasitic amphipods, and although there was no evidence that the penguins were targeting these, they may prove to be a profitable addition (Thiebot et al. 2016).

Two species of Scyphozoa were measured from both the winter Weddell Sea (PS81) and the summer Lazarev Sea (PS89) using bomb calorimetry (Table 10). The energetic density of *Periphylla periphylla* was 20.4 kJ g^−1^ DW during winter. Samples consisted of one small individual (93.5 g WW) and several larger individuals, with a WW ranging from 470 to 499 g. The average winter energy density of *P. periphylla* was higher compared to 10.8 kJ g^−1^ DW during summer. The latter measurements were, however, performed on small individuals with an average WW of 7.0 g. This suggests that season has an influence on the energy density of *P. periphylla*, although there could also be an influence of size. No difference was found in the energy density of *Atolla* spp. between seasons. The average energy density of *Atolla* spp. was 11.0 kJ g^−1^ DW during winter and 12.3 kJ g^−1^ DW during summer. The water content of the Scyphozoa caught during both winter and summer was similar and usually in between 90 and 95% WW. It should be kept in mind that these individuals were weighed after having been frozen. Due to the potential error in that measurement, energy densities are not given in kJ g^−1^ WW. High ash contents may have resulted in an underestimation of the dry weight energy densities of these Scyphozoa.

**Table 10:** Average energy density of scyphozoans ± standard deviation

Season	Location	*n*	Mean DW (mg)	Water content (%)	Energy density (kJ g^−1^ DW)	Method	Source
*Periphylla periphylla*							
Winter/spring	Weddell Sea	8	22.0 ± 9.1	93.6 ± 1.7	20.42 ± 1.13	BC	This study (PS81)
Summer	Lazarev Sea	9	0.8 ± 0.9	89.0 ± 6.1	10.85 ± 2..57	BC	This study (PS89)
*Atolla* sp.							
Winter/spring	Weddell Sea	5	1.57 ± 0.7	93.0 ± 2.7	11.16 ± 3.79	BC	This study (PS81)
Summer	Lazarev Sea	16	1.01 ± 0.3	93.2 ± 1.6	12.29 ± 1.41	BC	This study (PS89)

Measurements were done using bomb calorimetry. *n* represents the number of samples measured

Observations showed that Adélie penguins often attacked the gonads and/or oral arms of jelly fish specifically, and that there was a relationship between the penguin attacks and the visible presence of gonads (Thiebot et al. 2016). Gonads from *P. periphylla* caught in the summer Lazarev Sea showed a higher energetic density than other body parts (Table 11). Doyle et al. (2007) and Milisenda et al. (2014) also found that gonads had a higher energy content than oral arm or bell tissue, with the exception of one species in which the oral arms yielded a similar energy density as the gonads (Doyle et al. 2007). The energy densities of the bell and collar tissue of *P. periphylla* were very low and likely unrealistic (Table 11; Doyle et al. 2007). These tissues also had very high ash contents (Table 11), although ash content was high in general when compared to other animals.

**Table 11:** The energy density ± standard deviation of different body parts from the scyphozoan *Periphylla periphylla*, caught in the summer Lazarev Sea

	Mean WW (g)	Mean DW (g)	Water content (%)	Ash (% DW)	Energy density	
					kJ g^−1^ DW	kJ g^−1^ AFDW
Intestine	385.27	18.31	95.25	66.27 ± 0.39	6.73 ± 0.27	19.96
Gonads	113.12	7.66	93.23	41.57 ± 2.52	13.28 ± 0.12	22.73
Bell	94.46	3.66	96.12	74.90 ± 0.93	1.15 ± 0.28	4.59
Tentacles	123.02	5.61	95.44	55.89 ± 4.47	8.06 ± 2.32	18.27
Collar	259.09			75.30 ± 0.51	1.47 ± 0.33	5.97

Measurements were done using bomb calorimetry. Replicate measurements were performed on the body parts of a single individual

A measurement using bomb calorimetry on a sample of pooled anterior nectophores of the siphonophore *Diphyes antarctica* from winter Weddell Sea (PS81) resulted in an energy density of 12.0 kJ g^−1^ DW (4.0 kJ g^−1^ WW). The ash content of *D. antarctica* has been reported to be close to 60% (Donnelly et al. 1994).

Proximate compositions of ctenophore and cnidarian species were measured by Clarke et al. (1992) and Donnelly et al. (1994), which included the species *Beroe* spp. (Clarke et al. 1992), *Pleurobrachia* sp. (Clarke et al. 1992), *Calycopsis borchgrevinki* (Clarke et al. 1992; Donnelly et al. 1994), *Botrynema brucei* (Clarke et al. 1992), *Diphyes antarctica* (Clarke et al. 1992; Donnelly et al. 1994) *P. periphylla* (Donnelly et al. 1994) and *Atolla wyvillei* (Clarke et al. 1992; Donnelly et al. 1994). The water content of all species was > 95% WW, while the ash content ranged between 50 and 73% DW (Clarke et al. 1992; Donnelly et al. 1994). Apart from residual water, there is evidence that suggests that gelatinous species also contain a proportion of amino-carbohydrate which is missed by conventional assay techniques. Furthermore, a proportion of the protein potentially consists of glycoproteins that can be missed or underestimated depending on the technique used (Clarke et al. 1992). This could explain why energy density calculated using proximate composition is far lower than the energy density of carbohydrates, and is an unreliable method for estimating energy density of gelatinous species (Clarke et al. 1992; Donnelly et al. 1994).

Pelagic tunicates, or salps, that occur in the Southern Ocean are widely distributed and can form an important part of the total metazoan biomass, particularly in relatively warm water masses (Pakhomov 2004; Pakhomov et al. 2011). The proximate composition of the pelagic tunicates *Salpa fusiformis*, *Salpa thomsoni* and *Ihlea racovitzai* were measured by Clarke et al. (1992), Donnelly et al. (1994), Dubischar et al. (2006) and Dubischar et al. (2012). Despite similar complications as for other gelatinous zooplankton, some of the sources report an energy density estimate. Dubischar et al. (2012) estimated the WW energy density of *S. thompsoni* and *I. racovitzai* to be 0.2 and 0.4 kJ g^−1^ (using the conversion factors 4.1 kcal g^−1^ for protein and 9.3 kcal g^−1^ for lipids), which would correspond to 3.1 and 6.7 kJ g^−1^ DW, respectively. They did find that the energy density of *I. racovitzai* was approximately twice as higher than that of *S. thompsoni*, mainly due to differences in the amount of protein. The amount of protein found by Donnelly et al. (1994) was a lot lower. The proximate composition did not markedly differ between seasons in both studies, suggesting that lipids are not accumulated (Donnelly et al. 1994; Dubischar et al. 2012). Clarke et al. (1992) calculated an energy density of 4.6 kJ g^−1^ DW for *S. fusiformis*. When comparing solitary forms with aggregate forms of *S. thompsoni* measured from the Bellinghausen Sea in autumn, the amount of protein and lipids was higher in the former, which would result in a higher energy density for the solitaries when converted (5.3 kJ g^−1^ DW as opposed to 3.2 kJ g^−1^ DW; Dubischar et al. 2006). The reported energy densities for salps are also lower than that of carbohydrates (Clarke et al. 1992). Questions still remain regarding the digestibility of salps. It is suggested that they can be digested entirely but also only partly due to the cellulose-like tunicin present in the tunica (Dubischar et al. 2012 and references therein). Gili et al. (2006) proposed that the salps’ stomach may be the main source of energy when preyed upon.

#### Chaetognaths, polychaetes and gastropods

Other pelagic zooplankton species for which reported energy densities were found, included chaetognaths, polychaetes and a gastropod (Table S4 of the electronic supplement). Chaetognaths, such as *Eukrohnia hamata*, *Sagitta gazellae* and *Sagitta marri*, can form a major part of the mesopelagic zooplankton community and are important carnivorous predators (Pakhomov et al. 1999; Flores et al. 2014). These three species are the most abundant in the epipelagic and have a wide, circumpolar distribution (David 1958). Their distribution in the water column has been found to follow increased abundances of their prey, larval krill and copepods (David et al. 2017).

Estimated energy density using proximate composition is available for the three species of chaetognaths and two species of polychaetes in Donnelly et al. (1994). The dry-weight energy density of chaetognaths ranged between 5.0 kJ g^−1^ DW of *S. gazellae* caught in autumn and 11.7 kJ g^−1^ DW of *E. hamata* caught during winter. The energy densities of *E. hamata* and *S. gazellae* were higher in winter than in autumn. Seasonal changes in energy content are suggested to be a result of trophodynamics (Donnelly et al. 1994). *Sagitta marri* had a winter energy density of 11.3 kJ g^−1^ DW. All chaetognath species had high water and ash contents.

The polychaetes *Vanadis antarctica* and *Tomopteris carpenteri* are both oceanic species that can be found in the entire water column (Boysen-Ennen and Piatkowski 1988; Fernández-Álamo and Thuesen 1999). They had estimated energy densities of 14.2 kJ g^−1^ DW during autumn and 9.2 kJ g^−1^ DW during winter, respectively (Donnelly et al. 1994). The energy density of *T. carpenteri* was also estimated from individuals caught near South Georgia. The reported value of 12.4 kJ g^−1^ DW was calculated using the values 39.5 kJ g^−1^ for lipids and 23.9 kJ g^−1^ for protein (Clarke et al. 1992).

A proximate composition estimate of the pelagic gastropod *Clione limacina antarctica* from the McMurdo Sound yielded an energy density of 24.8 kJ g^−1^ DW (Bryan et al. 1995). This gastropod can be very abundant in certain seasons or areas, and contains defensive chemicals to defend itself against predation (Bryan et al. 1995).

#### Benthic invertebrate species

McClintock (1987, 1989) and McClintock et al. (2004, 2006) reported energy densities of benthic echinoderms, sponges and a tunicate. All estimates of the energy density of these species were done using proximate composition. The sea stars *Granaster nutrix* and *Neosmilaster georgianus* were investigated in McClintock et al. (2006). Measurements were done on the pyloric caeca and body wall separately which yielded 24.8 and 8.5 kJ g^−1^ DW for *G. nutrix*, and 26.5 and 14.1 kJ g^−1^ DW for *N. georgianus*, respectively. The energy densities of the body walls of 13 echinoderm species from McMurdo Sound ranged from 10.5 (*Odontaster meridionalis*) to 18.2 (*Porania antarctica*) kJ g^−1^ DW (McClintock 1989). The proximate composition of different body parts of the aforementioned study can be found in McClintock and Pearse (1987). The energy densities of 17 species of benthic sponges from McMurdo Sound ranged from 5.1 kJ g^−1^ DW (*Sphaerotylus antarcticus*) to 17.4 kJ g^−1^ DW (*Dendrilla membranosa*; McClintock 1987). The energy density of the benthic tunicate *Distaplia cylindrica* was estimated at 14.7 kJ g^−1^ DW (McClintock et al. 2004). This benthic tunicate had a lower water content and higher protein content compared to the pelagic tunicates (Donnelly et al. 1994; McClintock et al. 2004; Dubischar et al. 2012).

An energy density of 21.8 kJ g^−1^ DW was estimated using proximate composition for the nemertean *Parborlasia corrugatus*, collected in the McMurdo Sound during spring (Heine et al. 1991). Three bivalve species from the Patagonian shelf, *Aulacomya atra*, *Perumytilus purpuratus* and *Mytilus edulis*, yielded energy densities of 19.2, 20.0 and 17.9 kJ g^−1^ DW, respectively. Animals were measured without shells using bomb calorimetry (Ciancio et al. 2007).

## Discussion

### Data gaps

There is a focus on certain species, but the Southern Ocean is composed of different biogeographical regions that can have a distinct biodiversity and community structure, and specific key species. For instance, for euphausiids many studies focus on *Euphausia superba*, but other species can be of high importance in certain areas. In the continental shelf region, *Euphausia crystallorophias* is an important food source, for instance for Adélie penguins in the Ross Sea and at Adélie Land (Ainley et al. 1998; Cherel 2008), and for minke (*Balaenoptera acutorostrata*) and blue whales (*Balaenoptera musculus*; Laws 1977; Ishii et al. 1998). The euphausiid *Euphausia vallentini* can be a major food source for many sea birds in particular biogeographic regions, for instance at Heard Island and the Crozet Islands (Ridoux 1994; Deagle et al. 2007). Several amphipod species are found in the diet of many bird species (Hindell 1989; Ridoux 1994; Van Franeker et al. 2001), although their contribution to the diet varies significantly between regions. The hyperiid amphipod *Themisto gaudichaudii*, for example, has a wide distribution and is found in variable amounts in the diet of many species, but seems to be an important prey item in the region of the Kerguelen Islands particularly (Bocher et al. 2001). Similarly, the copepod *Paraeuchaeta antarctica* has been found to be abundant in the diet of bird species in the Kerguelen region (Bocher et al. 2002).

A better seasonal or regional coverage of the energy density of species is desirable as it can give insight into a species life cycle and behaviour, because several top predator species show a change in diet between seasons (Ridoux 1994). For example, the fish feeding Cape petrel (*Daption capense*) switches to a squid dominated diet in the Weddell/Scotia Sea in autumn (Ainley et al. 1991), while the Arctic tern (*Sterna paradisaea*) feeds mainly on *Electrona antarctica* in spring, but on Antarctic krill in autumn (Ainley et al. 1991). Adélie penguins in the Ross Sea, feeding mainly on krill at the start of the season, increased their proportion of fish in the diet together with their foraging trip duration. This is likely a result of a change in food availability due to increased predation pressure by the penguins themselves (Ainley et al. 2015).

There are many species groups that are overlooked as they are not known to be an important part of the diet of top predators, but which can reach high numbers and biomasses in certain habitats or seasons and are, therefore, important parts of the Southern Ocean food web. These include previously mentioned groups such as salps (Pakhomov et al. 2002), chaetognaths, siphonophores, ctenophores, gastropods (Hunt et al. 2008; Flores et al. 2011, 2014), other small krill species such as *Euphausia frigida*, but also benthic species such as bivalves and limpets (Favero et al. 1997; Ainley et al. 2003). Furthermore, a better coverage of the energy density of Southern Ocean species can help to predict what happens if prey distribution changes. For example, research has shown that areas dominated by Antarctic krill may be replaced with a dominance of salps due to warming waters (Pakhomov et al. 2002; Atkinson et al. 2004; Ross et al. 2014), which may have significant food web implications. An effect of food availability on annual fledging mass of Macaroni penguin chicks (*Eudyptes chrysolophus*) was shown at Bird Island, South Georgia (Waluda et al. 2012). The fledging mass of penguin chicks could be related to the energy density of the prey in combination with prey size and mobility, and was highest in years where *E. superba* dominated the diet, and lowest when there were large proportions of fish and other crustaceans, such as *T. gaudichaudii* and *E. frigida* (Waluda et al. 2012). In contrast, male Adélie penguin chicks had a higher proportion of fish in the diet and were growing faster than female chicks, which ate higher proportions of krill (Jennings et al. 2016). Model simulations also suggested that penguin chicks that supplemented their diet with fish (*Pleuragramma antarctica*), instead of feeding solely on Antarctic krill, would be heavier and more likely to recruit (Chapman et al. 2011). The quantity of milk fat of fur seals (*Arctocephalus gazella*) at Kerguelen was found to be influenced by the proportion of myctophids in the diet (Lea et al. 2006).

The energy density of a prey species might change as consequence of warming temperatures. Oxygen consumption and metabolic rate have been found to increase with increasing temperature across species (Brockington and Clarke 2001). This could not only lead to smaller sized individuals (Atkinson 1994; Daufresne et al. 2009; Baudron et al. 2014), but also to changes in community structure due to a need for increased consumption leading to, for example, changes in predator–prey interactions or intraspecific competition (Bruno et al. 2015). The reduction in body size with increasing temperature has been found for many myctophid fish species, which could potentially lead to these fish shifting to a different size of prey or becoming a less valuable food source for predators (Saunders and Tarling 2018). In addition, the energy allocation (for instance, towards growth or build-up of reserves) has been found to change under different temperature conditions in a study on zoarcid fish species (Brodte et al. 2006).

Although for all types of studies using a species-specific energy density value it would be preferable to use an estimate that is specific for, e.g. region, season and body size, a generalized estimate of the energy density of a species could be useful in cases where this is not available. For many species, however, only a single record of their energy density exists. Many records also often consist of one individual or a single-pooled sample. Therefore, more measurements are necessary to validate and generalize energy densities of species, and sources of variation within species. For *E. antarctica* there are relatively many individual records (284), which yield a mean energy density of 30.26 kJ g^−1^ DW and 8.94 kJ g^−1^ WW. Results have shown, however, that sources of variation include size and region. Another way to estimate a mean value could be using a median value of all recorded mean energy densities, which would result in median values of 29.61 kJ g^−1^ DW and 9.08 kJ g^−1^ WW for *E. antarctica* and 21.9 kJ g^−1^ DW and 5.01 kJ g^−1^ WW for *E. superba*. For the latter species it is, however, clear that the energy densities differ between sexes and developmental stages, while regional differences are uncertain. For aforementioned estimates only bomb calorimeter measurements were used.

Measuring energy density using bomb calorimetry and proximate composition are time consuming. Therefore, increased information on relationships between energy density and other, more easy to measure, parameters could be helpful. These may include insights in the effect of size/age/maturity on energy density within species and variation between seasons and regions. Also, relationships between water content or proportion of body carbon and energy density, including more information on differences and similarities between, for example, species, families and classes, would be useful to evaluate the accuracy of values used. In addition, it would increase the precision of studies and models based on energy density, when using values that take interspecific variation into account. Currently, regressions are generally limited to certain fish species and on an individual basis for Antarctic krill (Färber-Lorda et al. 2009a). To obtain such correlations, measurements on individuals are most useful. A standard bomb calorimeter needs, however, quite a large dry weight sample and thus for measuring small animals it is necessary to have access to a micro-bomb calorimeter.

### Size/age–energy density relationships

Relationships between size and dry-weight energy density are found for fish, but differ between species. A positive relationship between size and dry weight energy density was found for the myctophids *Gymnoscopelus piabilis*, *Electrona carlsbergi* (Lea et al. 2002) and *E. antarctica*. For other fish species such as *Bathylagus antarcticus* (Tierney et al. 2002; Van de Putte et al. 2006), *Pleuragramma antarctica* (Van de Putte et al. 2010) and other fish from the study of Lea et al. (2002), no relationship was found, and fish had the same dry-weight energy density regardless of size. In addition, Tierney et al. (2002) found negative relationships between size and dry weight energy density. Most relationships are, however, not linear, but show differences between size classes. Therefore, as recommended by Van de Putte et al. (2006), it is useful to separate energy densities in age or size classes, using distinct energy densities for each age group. In particular, in trophodynamic studies and research on prey utilization of species, as predators are known to often feed on a particular prey size (Van Franeker et al. 2001). However, again more data are needed to see if size/energy density relationships show a general trend rather than an incidental occurrence, and if differences are found, to be able to characterize the size classes between which differences occur. The available data on *Gymnoscopelus braueri* show an example where there is a (negative) relationship in one dataset, but none in the other (Tierney et al. 2002; Van de Putte et al. 2006). Furthermore, it is currently unclear how energy density in young fish is allocated because not all small specimens show increased energy density with decreasing water content, as would be expected (Tierney et al. 2002).

In krill, and most likely other crustaceans, there are marked differences in energy density between developmental stages. Predators have been found to have a higher proportion of female krill in their diet, probably also due to their larger size (Reid et al. 1996). It would be useful to gain information on the energy densities of krill based on size, as predators also prey upon particular sizes, and sizes of different developmental stages usually overlap. For instance, fulmarine petrels consume krill of approximately 35 mm (Van Franeker et al. 2001), which is a size including both juvenile and sub-adult krill (Siegel 1987, 2012). For species other than *E. superba* and fish, size or developmental stage specific data are lacking completely, although data suggest that there may be differences in energy density between size classes, for example in the jelly fish *Periphylla periphylla*.

### Water content–energy density relationships

The relationship between water content and energy content (in kJ g^−1^ WW) can help estimating the energy density based on water content (usually expressed in DW as a percentage of WW), in which case only the determination of wet weight and dry weight or water content is needed (Hartman and Brandt 1995). The relationship between water content and energy density (WW) of *E. antarctica* was similar between seasons and regions, and thus a single-regression model, using all available individual measurements, should give good, generally useful parameters for the estimation of energy density on a WW basis given the water content:

$${\text{ED}}_{\text{WW}} = 0.393 \times P_{\text{DW}} {-}2.977\;(R^{2} = \;0.93,\;n = 252),$$

where ED_WW_ represents the energy density in kJ g^−1^ WW and *P*_DW_ the dry weight as a percentage of WW.

Using the available individual data of *G. braueri*, a generalized regression model would yield similar parameters:

$${\text{ED}}_{\text{WW}} = 0.344 \times P_{\text{DW}} {-}1.539\;(R^{2} = 092,\;n = 33).$$

This model does, however, exclude the smaller fish (< 40 mm) from Tierney et al. (2002) which had a significantly higher energy density than the larger fish, for which the cause remains unclear. The slopes of the models for *E. antarctica* and *G. braueri* differed significantly from each other (ANCOVA, *p* = 0.006).

Differences in regression slopes between fish species (Van de Putte et al. 2006, 2010) reveal that the relationship between water content and energy density (WW) differ between families at least. Hartman and Brandt (1995) suggested that similar models can be used for fish within the same order or family, but recommended using species-specific models when available, especially in species which show marked seasonal changes in energy density. In this review, individual data on *Bathylagus antarcticus* showed that the relationship can also differ between seasons and or regions. Furthermore, the different feeding habitats and wide range of energy densities of nototheniid fishes suggest that there might be large differences in water content/energy density relationships between species of the same family. Similar modelling was done by Ciancio et al. (2007), including crustaceans, fish and cephalopods. They also found that same genus models would produce similar results as species-specific models, although this was not the case for some groups which were less well represented by aggregated models and for which species-specific models were recommended. Therefore, more individual data are needed to establish regression models for different species, to compare the relationship between water content and energy density within families and to evaluate if the established regression models can be used in a general manner, also for taxa other than fish.

## Conclusion

A large amount of data are available on the energy density of potential prey species in the Southern Ocean. The available data are, however, strongly skewed towards a few large, abundant and relatively easily accessible taxa. Furthermore, information on the seasonal and regional variability of energy densities is still limited in most species. This information, however, would be key to the improvement of bio-energetic models and food web models. Bomb calorimetry is hitherto regarded as the most accurate method for energy density measurements. However, proximate composition analysis at various levels can provide a range of additional parameters often used in ecological studies. Important taxa for the energy flux of Antarctic food webs remain under-sampled. In a changing Southern Ocean, smaller zooplankton and gelatinous species may become more abundant. Such a shift would likely change food web energetics significantly at various levels, affecting the carrying capacity of the ecosystem for top predators and harvesting of living resources. It will, therefore, become increasingly important to include small and gelatinous zooplankton in energy flux models and ecosystem studies, warranting the need for more energetic measurements of these organisms.

## Electronic supplementary material

Below is the link to the electronic supplementary material.

Supplementary material 1 (PDF 457 kb)
