# Supplementary material for: Review: the energetic value of zooplankton and nekton species of the Southern Ocean
Source: Mar Biol. 2018 Jul 18;165(8):129. doi: 10.1007/s00227-018-3386-z (PMC6061478; doi:10.1007/s00227-018-3386-z)
Supplement: Supplementary file 1 — Supplementary material 1 (PDF 457 kb) [file 227_2018_3386_MOESM1_ESM.pdf]

Electronic supplement: overview tables of the energy densities of copepods, amphipods, other crustacea, chaetognaths, polychaetes and gastropods of the Southern Ocean.

Article title: Review: the energetic value of zooplankton and nekton species of the Southern Ocean

Journal: Marine Biology

Authors: F.L. Schaafsma, Y. Cherel, H. Flores, J.A. van Franeker, M.A. Lea, B. Raymond, A.P. van de Putte

Affiliation: Wageningen Marine Research , Ankerpark 27, 1781 AG Den Helder, The Netherlands.

E-mail address: fokje.schaafsma@wur.nl

Table S1. Overview of the average energy density of copepod species from Donnelly et al. (1994). All values were estimated using proximate composition (PC). *n* represents the number of samples measured. Where this expresses samples of pooled individuals, this is indicated with (p). The standard deviation is given where available (±).

Season	Location	*n*	Mean WW (g)	Water Content (%)	Mean energy density kJ g^{-1} WW	kJ g^{-1} DW	Method	Source
Calanoides acutus								
Autumn	Weddell Sea	2 (p)	0.20	86.0	2.51	17.57	PC	Donnelly et al. 1994*
Winter	Scotia Sea	2 (p)	0.78	84.2 ± 0.1	2.51	15.48	PC	Donnelly et al. 1994
Calanus propinquus								
Autumn	Weddell Sea	2 (p)	0.19	74.0	5.44	21.34	PC	Donnelly et al. 1994
Winter	Scotia Sea	2 (p)	0.49	84.6 ± 0.5	2.09	13.39	PC	Donnelly et al. 1994
Euaugaptilis laticeps								
Autumn	Weddell Sea	1 (p)	0.04	83.7	1.67	10.04	PC	Donnelly et al. 1994
Paraeuchaeta antarctica								
Autumn	Weddell Sea	1 (p)	0.22	79.3	4.60	21.76	PC	Donnelly et al. 1994
Winter	Scotia Sea	2 (p)	0.33	84.2 ± 1.8	3.35	20.08	PC	Donnelly et al. 1994
Euchirella rostromagna								
Winter	Scotia Sea	1 (p)	0.15	84.5	1.26	9.20	PC	Donnelly et al. 1994
Gaetanus tenuispinus								
Autumn	Weddell Sea	1 (p)	0.25	85.0	1.67	12.13	PC	Donnelly et al. 1994
Winter	Scotia Sea	3 (p)	0.19	82.6 ± 0.9	2.09	12.13	PC	Donnelly et al. 1994
Heterorhabdus austrinus								
Winter	Scotia Sea	1 (p)	0.22	88.7	1.26	12.13	PC	Donnelly et al. 1994
Heterorhabdus farrani								
Winter	Scotia Sea	1 (p)	0.17	89.5	1.26	12.13	PC	Donnelly et al. 1994
Metridia gerlachei								
Autumn	Weddell Sea	1 (p)	0.78	90.4	1.26	10.88	PC	Donnelly et al. 1994
Winter	Scotia Sea	1 (p)	0.46	91.0	0.84	9.62	PC	Donnelly et al. 1994
Rhincalanus gigas								
Autumn	Weddell Sea	1 (p)				12.55	PC	Donnelly et al. 1994
Winter	Scotia Sea	4 (p)	0.82	91.0 ± 0.3	1.26	13.81	PC	Donnelly et al. 1994

* A factor of 4.19 was used to convert calories to joules

Table S2. Overview of the average energy density of amphipod species. Values were estimated using proximate composition (PC) and one using bomb calorimetry (BC). Energetic values in italics represent values that were converted using information from the given source. *n* represents the number of samples measured. Where this expresses samples of pooled individuals, this is indicated with (p). The standard deviation is given where available (±).

Season	Location	*n*	Mean size (mm)	Water Content (%)	Mean energy density kJ g^{-1} WW	kJ g^{-1} DW	Method	Source
***Cyphocaris faueri* (gammarid)**								
Autumn	Weddell Sea	6	22.0	76.4 ± 5.8	2.42	*10.25*	PC	Torres et al. 1994*
***Cyphocaris richardi* (gammarid)**								
Autumn	Weddell Sea	5	28.8	74.7 ± 2.5	2.92	*11.54*	PC	Torres et al. 1994
Winter	Scotia Sea	5	22.6	74.8 ± 2.6	3.84	*15.24*	PC	Torres et al. 1994
***Parandania boecki* (gammarid)**								
Winter	Scotia Sea	2 (p)	18.5	83.7 ± 2.3	1.62	*9.94*	PC	Torres et al. 1994
***Eusirus microps* (gammarid)**								
Summer	Lazarev Sea	1 (p)	44.1 ± 1.7	80.67 ± 4.4	4.51	22.25	BC	This study (PS89)
***Cyllopus lucasii* (hyperiid)**								
Autumn	Weddell Sea	12	19.8	68.7 ± 4.2	5.69	*18.18*	PC	Torres et al. 1994
Winter	Scotia Sea	8	21.3	77.6 ± 2.1	2.87	*12.81*	PC	Torres et al. 1994
***Hyperia macrocephala* (hyperiid)**								
Autumn	Weddell Sea	1	30.0	72.8	3.77	*13.86*	PC	Torres et al. 1994
***Hyperiella antarctica* (hyperiid)**								
Autumn	Weddell Sea	1 (p)	9.6	86.7	1.71	*12.86*	PC	Torres et al. 1994
***Primno macropa* (hyperiid)**								
Autumn	Weddell Sea	2 (p)	14.3	70.6 ± 1.7	4.92	*16.73*	PC	Torres et al. 1994
Winter	Scotia Sea	2 (p)	14.7	76.5 ± 0.2	3.23	*13.74*	PC	Torres et al. 1994
***Themisto gaudichaudii* (hyperiid)**								
Winter	Scotia Sea	2 (p)	17.0	77.4 ± 0.5	2.88	*12.74*	PC	Torres et al. 1994
	Patagonia	3 (p)	3-12	86.0	3.11	*22.19*	BC	Ciancio et al. 2007
***Vibilia stebbingi* (hyperiid)**								
Autumn	Weddell Sea	1 (p)	11.5	71.4	4.11	*14.37*	PC	Torres et al. 1994
Winter	Scotia Sea	3 (p)	10.5	72.5 ± 5.2	3.83	*13.93*	PC	Torres et al. 1994

* A factor of 4.19 was used to convert calories to joules

Table S3. Overview of the average energy density of other crustacean species. All values were estimated using proximate composition (PC). Energetic values in italics represent values that were converted using information from the given sources. The mean size of the decapods and mysids represents the carapace length, for ostracods it represents the sphere diameter. *n* represents the number of samples measured. Where this expresses samples of pooled individuals, this is indicated with (p). The standard deviation is given where available (±).

Season	Location	*n*	Mean size (mm)	Water content (%)	Mean energy density kJ g^{-1} WW	kJ g^{-1} DW	Method	Source
Pasiphaea scotiae **(decapod)**								
Autumn	Weddell Sea	6	21.7	63.2 ± 2.7	8.40	*22.82*	PC	Torres et al. 1994*
Winter	Scotia Sea	8	21.3	63.3 ± 2.1	6.97	*19.00*	PC	Torres et al. 1994
Petalidium foliacium **(decapod)**								
Autumn	Weddell Sea	1	17.0	71.8	5.58	*19.77*	PC	Torres et al. 1994
Winter	Scotia Sea	3	13.3	67.4 ± 3.3	8.24	*25.27*	PC	Torres et al. 1994
Boreomysis rostrata **(mysid)**								
Winter	Scotia Sea	2	10.0	75.8 ± 0.9	4.40	*18.17*	PC	Torres et al. 1994
Eucopia australis **(mysid)**								
Winter	Scotia Sea	2	13.0	77.8 ± 1.8	5.32	*23.96*	PC	Torres et al. 1994
Gnathophausia gigas **(mysid)**								
Winter	Scotia Sea	4	16.8	69.4 ± 4.4	5.95	*19.43*	PC	Torres et al. 1994
Conchoecia antipoda **(ostracod)**								
Winter	Scotia Sea	1 (p)		87.8	1.67	11.72	PC	Donnelly et al. 1994*
Conchoecia belgicae **(ostracod)**								
Winter	Scotia Sea	1 (p)		85.9	1.26	7.95	PC	Donnelly et al. 1994
Conchoecia hettacra **(ostracod)**								
Winter	Scotia Sea	1 (p)		84.1	1.26	7.11	PC	Donnelly et al. 1994
Gigantocypros mulleri **(ostracod)**								
Winter	Scotia Sea	4	16.3	91.3 ± 0.4	0.70	*8.06*	PC	Torres et al. 1994

* A factor of 4.19 was used to convert calories to joules

Table S4. Average energy density of chaetognath, polychaete and a gastropod species. Energy densities in italics represent values that were converted using information from the given sources. *n* represents the number of samples measured. Where this expresses samples of pooled individuals, this is indicated with (p). Standard deviation is given were available. All measurements were done using proximate composition (PC).

Season	Location	*n*	Mean WW (mg)	Water content (%)	Mean energy density kJ g^{-1} WW	kJ g^{-1} DW	Method	Source
Eukrohnia hamata								
Autumn	Weddell Sea	1 (p)	0.455	95.0	0.42	7.53	PC	Donnelly et al. 1994*
Winter	Scotia Sea	1 (p)	1.998	91.8	0.84	11.72	PC	Donnelly et al. 1994
Sagitta gazellae								
Autumn	Weddell Sea	1 (p)	4.36	95.1	0.42	5.02	PC	Donnelly et al. 1994
Winter	Scotia Sea	3 (p)	1.36	93.5 ± 1.1	0.42	7.53	PC	Donnelly et al. 1994
Sagitta marri								
Winter	Scotia Sea	1 (p)	0.67	90.8	1.26	11.30	PC	Donnelly et al. 1994
Vanadis antarctica								
Autumn	Weddell Sea	1	1.09	86.3	2.09	14.23	PC	Donnelly et al. 1994
Tomopteris carpenteri								
Winter	Scotia Sea	1 (p)	0.757	87.7	1.26	9.20	PC	Donnelly et al. 1994
Summer	South Georgia	5		84.5	*1.92*	12.37[1]	PC	Clarke et al. 1992
Clione limacina antarctica								
Spring/summer	McMurdo Sound	4 (p)				*24.81*[2]	PC	Bryan et al. 1995

[1]calculated using the values 39.5 kJ g^{-1} for lipids and 23.9 kJ g^{-1} for protein

[2]calculated using 39.5 kJ g^{-1} for lipids

* A factor of 4.19 was used to convert calories to joules
